# Estimating SARS‐CoV‐2 infections and associated changes in COVID‐19 severity and fatality

**DOI:** 10.1111/irv.13181

**Published:** 2023-08-16

**Authors:** Valentina Marziano, Giorgio Guzzetta, Francesco Menegale, Chiara Sacco, Daniele Petrone, Alberto Mateo Urdiales, Martina Del Manso, Antonino Bella, Massimo Fabiani, Maria Fenicia Vescio, Flavia Riccardo, Piero Poletti, Mattia Manica, Agnese Zardini, Valeria d'Andrea, Filippo Trentini, Paola Stefanelli, Giovanni Rezza, Anna Teresa Palamara, Silvio Brusaferro, Marco Ajelli, Patrizio Pezzotti, Stefano Merler

**Affiliations:** ^1^ Center for Health Emergencies Bruno Kessler Foundation Trento Italy; ^2^ Department of Mathematics University of Trento Trento Italy; ^3^ Department of Infectious Diseases Istituto Superiore di Sanità Rome Italy; ^4^ Dondena Centre for Research on Social Dynamics and Public Policy Bocconi University Milan Italy; ^5^ COVID Crisis Lab Bocconi University Milan Italy; ^6^ Health Prevention directorate Ministry of Health Rome Italy; ^7^ Laboratory for Computational Epidemiology and Public Health, Department of Epidemiology and Biostatistics Indiana University School of Public Health Bloomington Indiana USA

**Keywords:** IFR, IHR, infection ascertainment ratio, infection fatality ratio, infection hospitalization ratio, SARS‐CoV‐2

## Abstract

**Background:**

The difficulty in identifying SARS‐CoV‐2 infections has not only been the major obstacle to control the COVID‐19 pandemic but also to quantify changes in the proportion of infections resulting in hospitalization, intensive care unit (ICU) admission, or death.

**Methods:**

We developed a model of SARS‐CoV‐2 transmission and vaccination informed by official estimates of the time‐varying reproduction number to estimate infections that occurred in Italy between February 2020 and 2022. Model outcomes were compared with the Italian National surveillance data to estimate changes in the SARS‐CoV‐2 infection ascertainment ratio (IAR), infection hospitalization ratio (IHR), infection ICU ratio (IIR), and infection fatality ratio (IFR) in five different sub‐periods associated with the dominance of the ancestral lineages and Alpha, Delta, and Omicron BA.1 variants.

**Results:**

We estimate that, over the first 2 years of pandemic, the IAR ranged between 15% and 40% (range of 95%CI: 11%–61%), with a peak value in the second half of 2020. The IHR, IIR, and IFR consistently decreased throughout the pandemic with 22–44‐fold reductions between the initial phase and the Omicron period. At the end of the study period, we estimate an IHR of 0.24% (95%CI: 0.17–0.36), IIR of 0.015% (95%CI: 0.011–0.023), and IFR of 0.05% (95%CI: 0.04–0.08).

**Conclusions:**

Since 2021, changes in the dominant SARS‐CoV‐2 variant, vaccination rollout, and the shift of infection to younger ages have reduced SARS‐CoV‐2 infection ascertainment. The same factors, combined with the improvement of patient management and care, contributed to a massive reduction in the severity and fatality of COVID‐19.

## 1 BACKGROUND

The COVID‐19 pandemic has been characterized by an ever‐changing epidemiological situation, forcing almost every country in the world to face a series of major challenges.^1^ During the first pandemic year, strict non‐pharmaceutical interventions (NPIs) were widely adopted to counter the spread of SARS‐CoV‐2 and prevent health care systems to be overwhelmed. These included social distancing restrictions culminating in nation‐wide lockdowns, school closures, and mandatory face masks.^2^, ^3^, ^4^, ^5^, ^6^ In Europe, the second pandemic year was characterized by a progressive relaxation of restrictions, the rollout of COVID‐19 vaccination campaigns,^7^ and, concurrently, by the emergence of new SARS‐CoV‐2 variants of concern.^8^, ^9^, ^10^, ^11^, ^12^

One of the main obstacles to pandemic control has been represented by underreported and underdiagnosed SARS‐CoV‐2 infections. Because of the difficulties in quantifying the extent of unobserved SARS‐CoV‐2 transmission over time, many aspects of the temporal changes in COVID‐19 epidemiology that occurred over the course of the pandemic remain unclear. Infection ascertainment ratios (IARs) likely changed over time because of the improvement in testing capacity, the increasing availability of diagnostics tests (also sustained by the development of quicker and cheaper antigen‐based detection technologies), the varying intensity of contact‐tracing, the shift of infections towards segments of the population less likely to develop symptoms, differences in pathogenicity associated with SARS‐CoV‐2 variants, the impact of external regulations (e.g., the requirement of a negative test result to access workplaces or community indoor spaces in absence of vaccination), and changes in the people's attitudes and behavior related to SARS‐CoV‐2 testing or in the self‐perception of symptoms associated with COVID‐19. If the actual number of SARS‐CoV‐2 infections is unknown, it is challenging to provide a solid estimate of the proportion of infections resulting in adverse outcomes (e.g., the infection fatality ratio [IFR]), which are crucial to inform the design and implementation of public health policies.

In this study, we propose a novel approach to quantify the daily number of SARS‐CoV‐2 infections in Italy, using a mathematical model of SARS‐CoV‐2 transmission informed with estimates of the time‐varying reproduction number Rt^13^ and data on COVID‐19 vaccine uptake.^14^ SARS‐CoV‐2 infections obtained through the model are then compared with integrated surveillance data^15^ to assess temporal changes in the IAR, the infection hospitalization ratio (IHR), the infection intensive care unit (ICU) ratio (IIR), and the IFR.

## 2 METHODS

**FIGURE 1:**
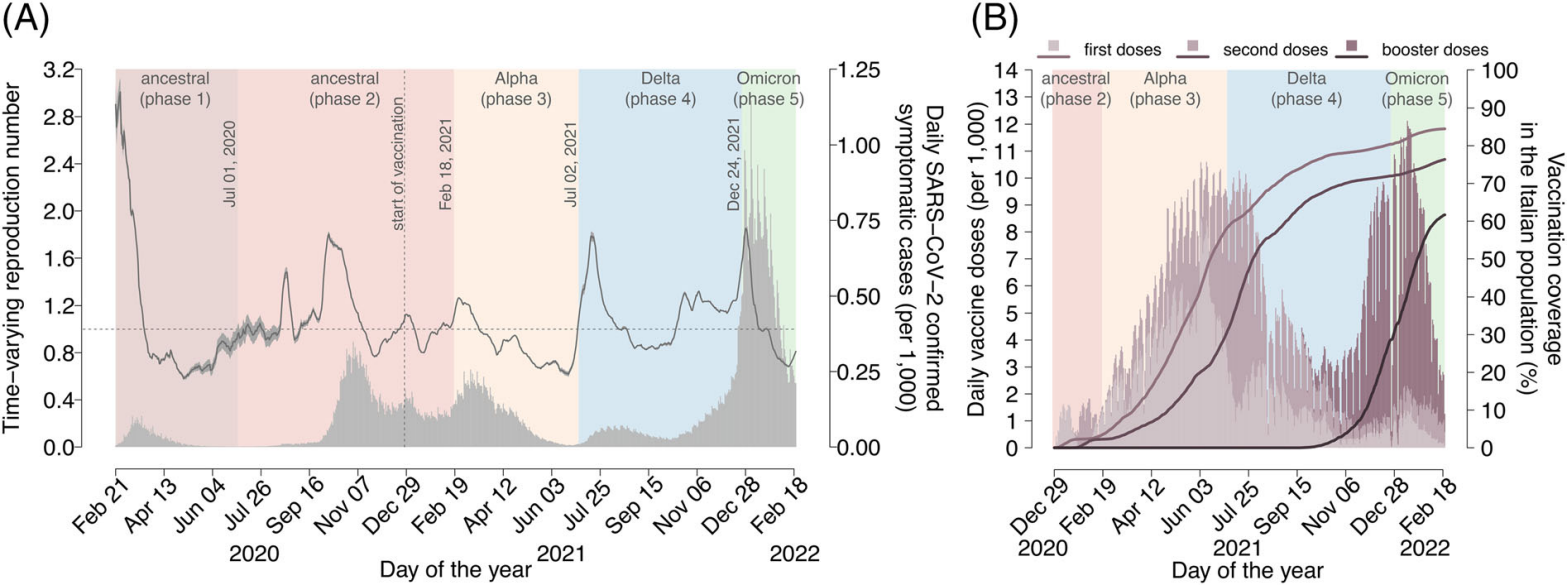
The COVID‐19 pandemic in Italy. (A) Mean estimates of the time‐varying reproduction number Rt as obtained from epidemic curves of symptomatic cases by date of symptom onset collected by the National Integrated Surveillance System^13^ (mean, grey solid line; shaded area, 95% CI; y‐axis on the left). Horizontal dotted line: epidemic threshold (Rt = 1). Grey bars represent the daily incidence per 1000 individuals of SARS‐CoV‐2 confirmed symptomatic infections by date of symptom onset as reported to the Italian Integrated Surveillance System^15^, ^18^ (y‐axis on the right). Background colors indicate the classification in different phases, and the dates indicated within the graph denote the day of transition between consecutive phases. The vertical dotted line denotes the start of the vaccination campaign on December 27, 2020. (B) Daily number of vaccine doses administered in Italy per 1000 individuals (stacked bar chart, y‐axis on the left).^14^ Line and bar colors, from lighter to darker shades, respectively indicate first, second, and booster doses. Solid lines show the cumulative vaccination coverage in the Italian population (y‐axis on the right). In Italy, by February 2022, administration of two doses was recommended to all individuals aged 5 years or more; administration of one booster dose was recommended to all individuals aged 12 years or more.

We developed an age‐structured stochastic model, based on a susceptible‐infectious‐removed‐susceptible (SIRS) scheme,^16^, ^17^ to simulate SARS‐CoV‐2 transmission and vaccination in Italy between February 21, 2020 (when the first locally transmitted case was detected) and February 20, 2022 (Figure S1). We divided the 2 years of simulation into five phases (background colors in Figure 1A). The first two phases are associated with the circulation of ancestral SARS‐CoV‐2 lineages, and they distinguish the first pandemic wave including the national lockdown (Phase 1, from February 21, 2020 to the end of June 2020) and a second phase characterized by a new upsurge of cases in fall 2020 and by the start of the COVID‐19 vaccination campaign on December 27, 2020 (Phase 2, from July 1, 2020 to February 17, 2021). The three remaining phases correspond to the periods of dominance of different SARS‐CoV‐2 variants in Italy: Alpha (Phase 3, from February 18, 2021 to July 1, 2021), Delta (Phase 4, from July 2, 2021 to December 23, 2021), and Omicron BA.1 (Phase 5, from December 24, 2021 to February 20, 2022).^19^ Conventional dates of transition between variants were chosen based on estimates of the prevalence of SARS‐CoV‐2 lineages from genomic surveillance data in Italy (Table S1).^19^ The model population is stratified by age (namely, 17 5‐year age groups from 0 to 84 years plus one age group for individuals aged 85 years or older). Mixing patterns across ages are assumed to be heterogeneous as estimated in the study of Mossong et al.^20^ At the beginning of the simulation, SARS‐CoV‐2 infection is seeded in a fully susceptible population, and the number of initially infectious individuals is determined in such a way to match COVID‐19 deaths reported by the surveillance system in the first ancestral phase (Figure S3).

The SARS‐CoV‐2 transmission rate on a given day was estimated in such a way that the model reproduction number (estimated via the Next Generation Matrix approach^21^, ^22^) matches the time‐varying reproduction number Rt as estimated from the epidemiological surveillance data (specifically, the number of new symptomatic cases by date of symptom onset, Figure 1A).^13^, ^15^, ^23^, ^24^

The model keeps into account the dynamics of age‐specific population immunity because of SARS‐CoV‐2 infection and vaccine uptake of the first, second, and booster doses (Figure 1B).^14^ In the model, individuals are considered eligible for vaccination, independent of a previous SARS‐CoV‐2 infection. Vaccine protection is assumed to be “leaky”, that is, successfully vaccinated individuals are partially immune with a relative risk of infection that depends on the SARS‐CoV‐2 variant and on the number of doses received.^25^, ^26^, ^27^ Breakthrough infections (i.e., infections in vaccinated individuals) are assumed to be half as infectious as those in unvaccinated individuals.^28^, ^29^

Natural infection provides complete protection against re‐infection with ancestral lineages and Alpha and Delta variants, whereas we assume a partial cross‐protection against Omicron BA.1 (set at 56%^30^). The case of a lower level of cross‐protection (set at 13%^31^) is explored in a sensitivity analysis (Table S4). Protection against re‐infection is assumed to wane exponentially after recovering from a natural infection with an average duration of 2 years over all periods considered.^32^ Alternative durations for the protection against re‐infection are explored for sensitivity analyses (1 and 10 years, Table S4). We consider waning of protection from vaccine‐induced immune response only in the Delta and Omicron phases.^25^, ^26^, ^27^ Waning of vaccine protection during the ancestral and Alpha phases is not considered; indeed, ancestral lineages were replaced by the Alpha variant a few weeks after the start of the vaccination campaign in Italy, and the waning of vaccine protection estimated in the literature during dominance of the Alpha variant is negligible.^26^, ^33^ Accordingly, we consider variant‐specific average durations of protection after two doses of vaccine and after a booster dose.^34^ Shorter and longer durations of vaccine protection are explored in the sensitivity analyses (Table S4). Further details on the model and parameter values are provided in the Supporting Information (Table S2).

**FIGURE 2:**
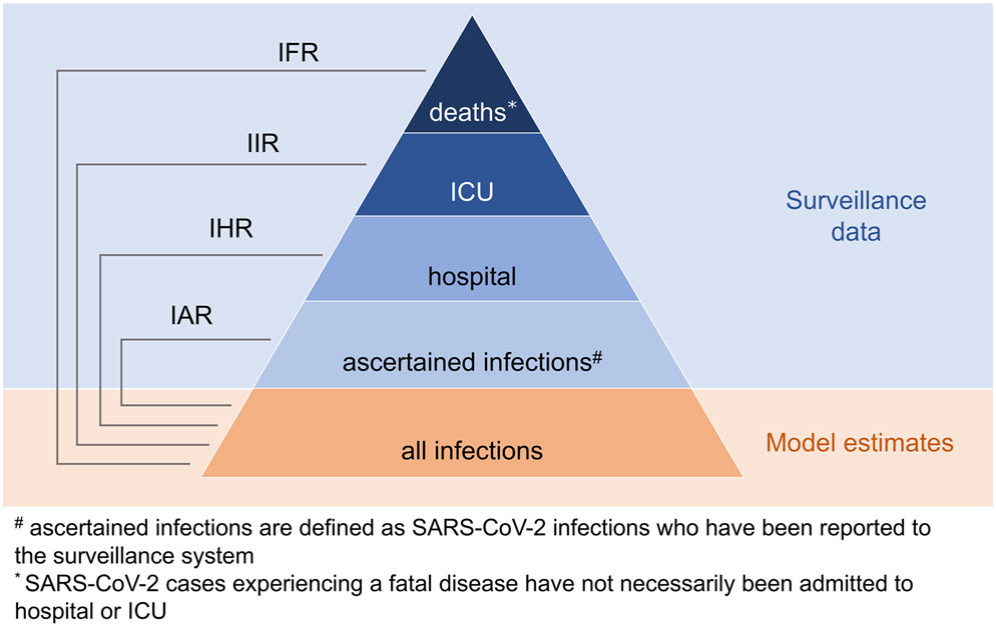
Schematic representation of the metrics of interest. Infection ascertainment ratio (IAR), ratio between SARS‐CoV‐2 ascertained infections and total infections estimated by the model; infection hospitalization ratio (IHR), ratio between hospitalized COVID‐19 cases and total infections estimated by the model; infection intensive care unit (ICU) ratio (IIR), ratio between COVID‐19 cases admitted to an ICU and total infections estimated by the model; infection fatality ratio (IFR), ratio between COVID‐19 deaths and total infections estimated by the model.

We estimated the SARS‐CoV‐2 IAR, IHR, IIR, and IFR as the number of SARS‐CoV‐2 ascertained infections, the number of hospitalized COVID‐19 cases, the number of COVID‐19 cases admitted to an ICU, and the number of COVID‐19 deaths, respectively, divided by the number of SARS‐CoV‐2 infections estimated by the model (Figure 2 and Table S3). All the numerators refer to numbers reported to the Italian Integrated Surveillance System.^15^ All the metrics were computed across the five considered phases, assigning reported infections to phases based on their date of diagnosis.

## 3 RESULTS

**FIGURE 3:**
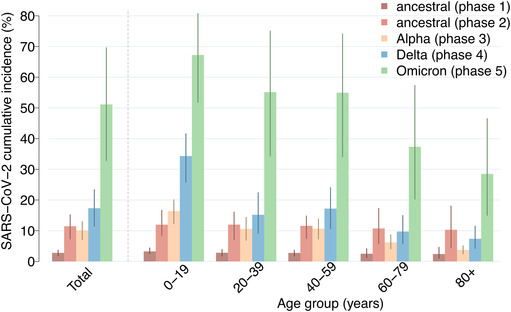
SARS‐CoV‐2 cumulative incidence. Estimated phase‐specific SARS‐CoV‐2 cumulative incidence (%) between February 21, 2020 and February 20, 2022 in the overall population and by age classes. Colors indicate the considered phases. Bars, mean estimates; vertical lines, 95% CI; *n* = 300 stochastic model realizations.

**FIGURE 4:**
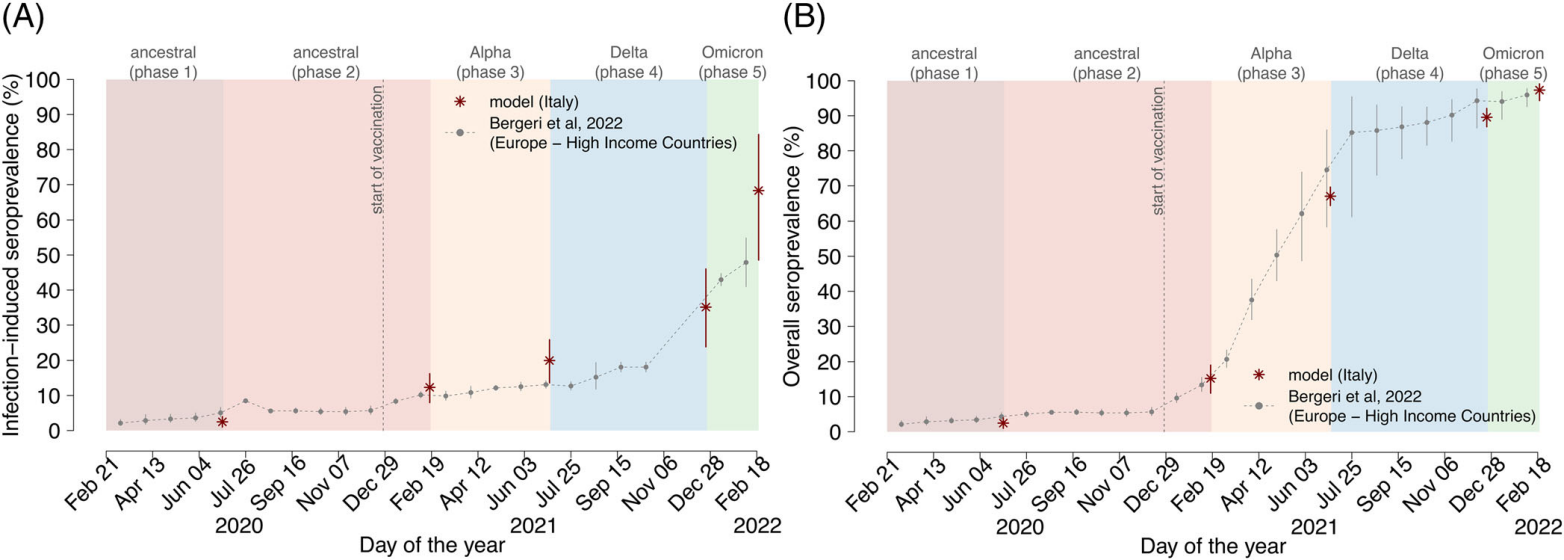
Validation of SARS‐CoV‐2 seroprevalence. (A) Grey dots represent weighted point estimates of the infection‐induced seroprevalence in high‐income European countries, as reported in a published meta‐analysis of population‐based serological studies.^35^ Red asterisks represent the mean proportion of the Italian population who had a previous natural infection, independently of vaccination status as estimated through the model. Vertical lines indicate 95%CI. (B) Grey dots represent weighted point estimates of the overall seroprevalence, that is, either induced by natural infection or vaccination, in high‐income European countries as reported in a published meta‐analysis of population‐based serological studies.^35^ Red asterisks represent the mean proportion of the Italian population who had a previous natural infection or has received one or more vaccine doses as estimated through the model. Vertical lines indicate 95%CI. Background colors indicate the classification in different phases, the vertical dotted line denotes the start of the vaccination campaign on December 27, 2020.

Despite the explosive spread of SARS‐CoV‐2 in the early phase of the pandemic, which threatened to overwhelm the Italian health system, we estimate that the adoption of a strict nationwide lockdown managed to limit the SARS‐CoV‐2 cumulative incidence in the first phase to 2.8% (95%CI: 1.8–3.6) (Figure 3). During the second phase, in the context of less stringent NPIs, we estimate a cumulative incidence of 11.4% (95%CI: 7.3–15.2). In both phases dominated by ancestral lineages, the SARS‐CoV‐2 cumulative incidence was substantially homogeneous across age groups (Figure 3). By mid‐February 2021, the ancestral SARS‐CoV‐2 lineages were replaced by the more transmissible Alpha variant,^8^ which remained dominant until early July 2021. During this period, we estimate that Alpha infected about 10.1% (95%CI: 7.1–13.0) of the Italian population, with a marked heterogeneity across ages: The highest cumulative incidence is estimated in the age group 0–19 years (16.4%, 95%CI: 12.3–20.1), whereas the lowest in people aged over 80 years (3.7%, 95%CI: 2.4–5.1) who had been prioritized for vaccination in the early months of 2021 (Figure 3 and Figure S2). The second half of 2021 was characterized by the circulation of the Delta variant, in the context of a progressive relaxation of NPIs, with an estimated cumulative incidence of 17.3% (95%CI: 11.4–23.4). Our results suggest that the progression of the vaccination campaign, including the administration of booster doses, led to a further shift of infections towards children and young adults, with over one third of infections occurring among individuals aged 20 years or less (Figure S4). By the end of December 2021, the Delta variant was replaced by Omicron BA.1. We estimate that, as of February 20, 2022, about 51.1% (95%CI: 32.8–69.6) of the Italian population had been infected with Omicron BA.1, with age‐specific cumulative incidence ranging from 28.5% (95%CI: 15.0–46.5) in individuals aged 80 years or more to 67.2% (95%CI: 51.8–80.7) in those younger than 20 years. Model estimates of the (infection‐induced and overall) seroprevalence of SARS‐CoV‐2 in Italy are consistent with population level seroprevalence estimates for high‐income European countries as obtained through a meta‐analysis of serological studies^35^ (Figure 4A,B).

**FIGURE 5:**
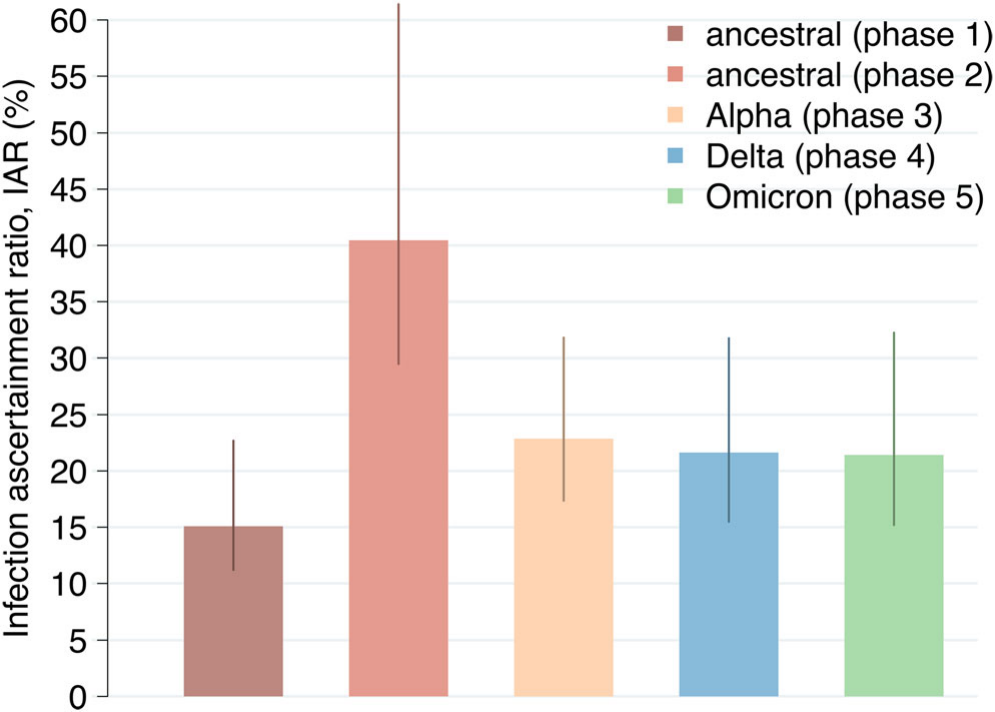
SARS‐CoV‐2 infection ascertainment ratio (IAR). Estimated phase‐specific SARS‐CoV‐2 infection ascertainment ratio between February 21, 2020 and February 20, 2022 (%). Bars: mean estimates; vertical lines: 95% CI; *n* = 300 stochastic model realizations.

We estimated an IAR of about 15.1% (95%CI: 11.2–22.7) in the first phase, corresponding to about one out of seven infections being detected by the Italian Integrated Surveillance System (Figure 5). In the second phase, we estimate a higher IAR of 40.5% (95%CI: 29.5–61.4). For the Alpha, Delta, and Omicron phase, we estimate a decrease of the IAR to 22.9% (95%CI: 17.3–31.8) in the Alpha phase, 21.6% (95%CI: 15.5–31.8) in the Delta phase, and 21.4% (95%CI: 15.2–32.2) in the Omicron phase.

**FIGURE 6:**
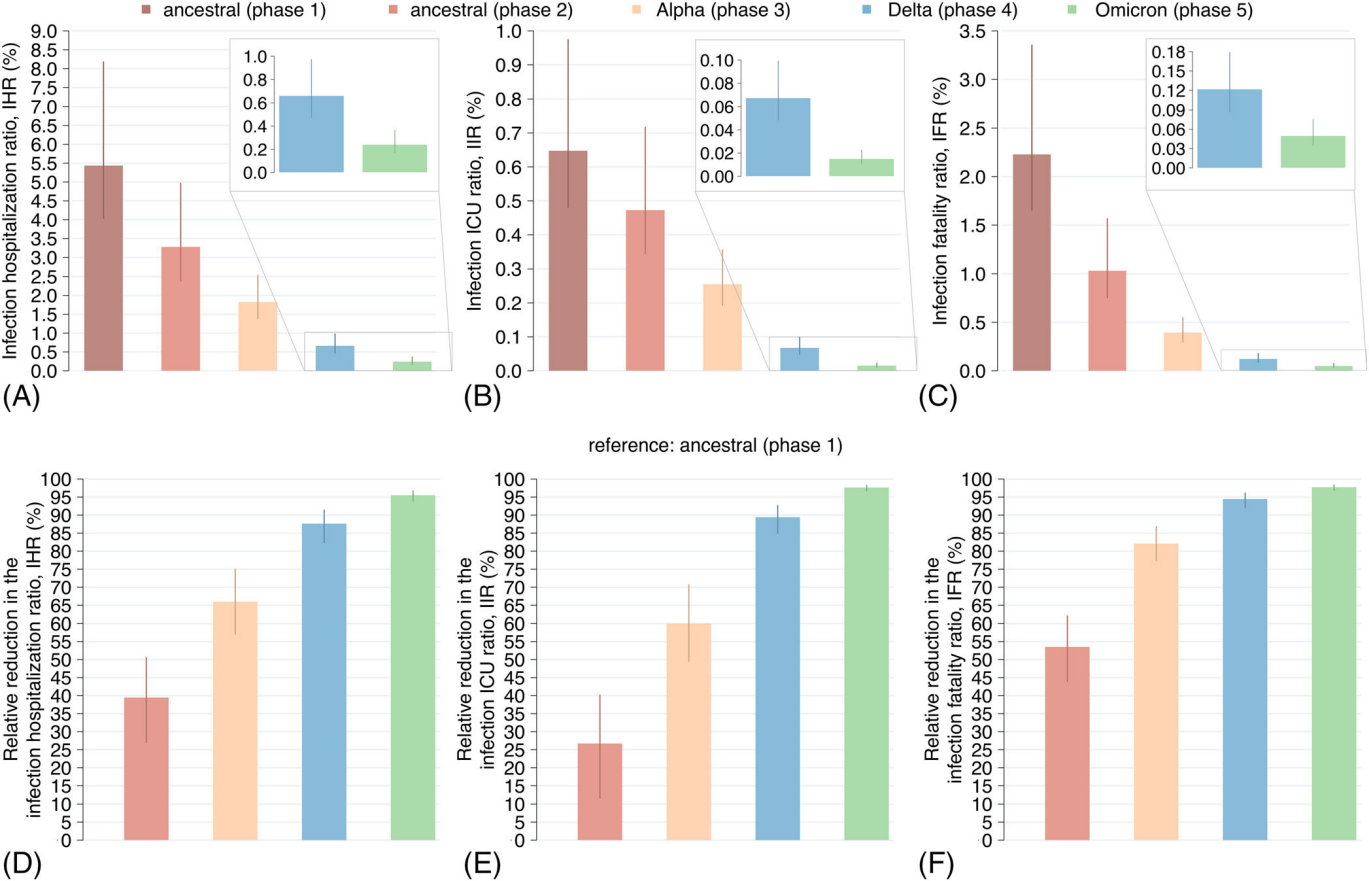
Changes in SARS‐CoV‐2 infection hospitalization ratio, intensive care unit (ICU) ratio and fatality ratio. (A) Infection hospitalization ratio (IHR). (B) Infection ICU ratio (IIR). (C) Infection fatality ratio (IFR). (D) Estimated relative reductions in the IHR compared with the first ancestral phase (%). (E) As D but for the IIR. (F) As D but for the IFR. Bars: mean estimates; vertical lines: 95% CI; *n* = 300 stochastic model realizations.

We estimated the IHR, IIR, and IFR in the different epidemic phases (Figure 6, first row). The first ancestral phase was characterized by the highest risk of developing severe clinical outcomes, with an IHR of 5.4% (95%CI: 4.0–8.2), an IIR of 0.65% (95%CI: 0.48–0.97), and an IFR of 2.2% (95%CI: 1.7–3.4). Estimates of all these ratios progressively decreased throughout the pandemic.

The IHR was estimated to decrease to 3.3% (95%CI: 2.4–5.0) in the second ancestral phase (39.5% reduction compared with first ancestral phase), 1.8% (95%CI: 1.4–2.5) in the Alpha phase (66% reduction compared with first ancestral phase; 43.9% compared with second ancestral phase), 0.66% (95%CI: 0.47–0.97) in the Delta phase (87.6% reduction compared with first ancestral phase; 63.7% compared with Alpha phase), and 0.24% (95%CI: 0.17–0.36) in the Omicron phase (95.5% reduction compared with first ancestral phase and 63.6% compared with Delta phase) (Figure 6A,D).

The IIR was estimated to decrease to 0.47% (95%CI: 0.34–0.72) in the second ancestral phase (26.7% reduction compared with first ancestral phase), 0.26% (95%CI: 0.19–0.35) in the Alpha phase (60% reduction compared with first ancestral phase; 45.5% compared with second ancestral phase), 0.07% (95%CI: 0.05–0.1) in the Delta phase (89.4% reduction compared with first ancestral phase and 73.6% compared with Alpha phase), and 0.015% (95%CI: 0.011–0.023) in the Omicron phase (97.6% reduction compared with first ancestral phase and 77.6% compared with Delta phase) (Figure 6B,E).

Finally, the IFR was estimated to decrease to 1.0% (95%CI: 0.8–1.6) in the second ancestral phase (53.5% reduction compared with first ancestral phase), 0.39% (95%CI: 0.3–0.55) in the Alpha phase (82.1% reduction compared with the first ancestral phase; 61.5% compared with second ancestral phase), 0.12% (95%CI: 0.09–0.18) in the Delta phase (94.5% reduction compared with first ancestral phase; 69.2% compared with Alpha phase), and 0.05% (95%CI: 0.04–0.08) in the Omicron phase (97.7% reduction compared with first ancestral phase; 59% compared with Delta phase) (Figure 6C,F). Estimates of the IHR, IIR, and IFR by age group show temporal trends compatible with the overall estimates (Figures S5–S7).

### 3.1 Sensitivity analyses

The results are only minimally affected by different assumptions on the duration of protection against re‐infection provided by immunity after natural infection (Figure S10–S13), on the duration of protection provided by vaccination (Figure S14–S17), and on the level of cross‐protection against Omicron BA.1 provided by infection with previous lineages (Figures S18 and S19).

We conducted a sensitivity analysis considering the same age‐specific relative susceptibility to SARS‐CoV‐2 infection in ancestral lineages and variants (as opposed to considering an age‐independent susceptibility for SARS‐CoV‐2 variants, as assumed in the main analysis). In particular, assuming a reduced susceptibility in children below 15 years and an increased in adults above 64 years resulted in a shift of infections towards older age groups compared with the main analysis in phases related to Alpha, Delta, and Omicron variants (Figure S20). Estimates obtained for the IAR, IHR, IIR, and IFR are, however, consistent with those obtained in the main analysis (Figures S20 and S21).

## 4 DISCUSSION AND CONCLUSIONS

In this work, we analyzed the first 2 years of the COVID‐19 pandemic in Italy to quantify changes in the SARS‐CoV‐2 IAR, IHR, IIR, and IFR, over five different pandemic phases associated to key events: the early phase, the second wave, and the periods of dominance of Alpha, Delta, and Omicron BA.1 variants.

The detection of SARS‐CoV‐2 infections changed throughout the pandemic. We estimated that the IAR increased from about 15% in the first ancestral phase, characterized by the first pandemic wave and the national lockdown, to 40% in the second ancestral phase, when the second pandemic wave occurred (Figure 5). Such increase is likely ascribable to the expansion of testing capacity (Figure S8), the strengthening of regional reporting systems, and an aggressive implementation of the test, track and trace strategy. In contrast, starting from the Alpha phase, the IAR stabilized to lower values around 20%. The decrease of the IAR with respect to the second ancestral phase is likely related to a combination of factors, including the availability of home testing leading to self‐diagnoses that were not notified to the surveillance system, the reduction in the frequency of contact tracing since 2021, and the significant increase of asymptomatic infections following the expansion of the vaccination program. Indeed, vaccination brought a shift of infections towards younger age groups (Figure S4) and increased the proportion of breakthrough infections (Figure S9); both trends reduced the overall probability of having symptoms given an infection,^36^, ^37^, ^38^ and therefore the overall probability of test‐seeking by unaware infected individuals.^39^

The large number of infections estimated during the Delta and Omicron phases may be ascribable to several factors, such as a possible decline in adherence to residual COVID‐19 restrictions due to pandemic fatigue^40^; the high transmissibility of these variants^8^, ^9^, ^10^, ^41^; the reduced efficacy of the vaccine in preventing infection for these variants^25^, ^26^, ^27^; the increased risk of reinfection during the Omicron BA.1 phase compared with previous epidemic phases^42^; the progressive release of restrictions, sustained by a lower morbidity among vaccinated individuals^37^, ^38^ and by a reduced intrinsic severity of Omicron.^43^, ^44^

Estimates obtained for the IHR, IIR, and IFR in the first phase of the pandemic (5.4%, 0.64%, and 2.2%, respectively) are in line with values reported in the literature for the pre‐vaccination period^45^, ^46^, ^47^, ^48^ (Figure 6). We found that the severity of SARS‐CoV‐2 infections has progressively declined throughout the pandemic, with the IFR in 2022 falling close to the levels of 2009 H1N1 pandemic influenza (estimated at about 0.02%^49^). Compared with the first pandemic wave, we estimated a 22‐fold lower IHR, 42‐fold lower IIR, and 44‐fold lower IFR during the Omicron BA.1 phase. The estimated decrease in the IFR in the post‐vaccination period is comparable to that reported in another study focusing on the Italian context.^50^ The estimated reduction in COVID‐19 severity is attributable to a combination of factors. Improved knowledge on the pathogen and patient management, and the relieving of the pressure on the health care system allowed by the national lockdown likely reduced severity between the first and second ancestral phases.^51^, ^52^ From the Alpha phase on, the vaccination program increased the population protection against severe disease.^25^, ^26^ In the absence of vaccination, the impact of SARS‐CoV‐2 variants could have been remarkably different.^17^, ^53^

Regarding the IIR, we note that our estimates do not necessarily reflect the probability of critical disease but may include the effect of patient management choices concerning trade‐offs between the usage of limited ICU resources and the expected benefits for the patient. In periods characterized by an intense pressure on the health‐care system and by a saturation of ICU bed occupancy,^52^ an expansion of ICU capacity may result in an increase of the IIR that does not necessarily correspond to an increase in the risk of critical disease.

The proposed model does not allow to disentangle the relative weight of individual determinants (e.g., vaccination, expanded therapeutic options, or variations in the intrinsic severity of SARS‐CoV‐2 variants) in the estimated reduction of the severity of SARS‐CoV‐2 infections.

In addition, estimates obtained for COVID‐19 severity rely on the assumption that all COVID‐19 cases who were hospitalized, admitted to ICUs, or died were reported to the Italian National Surveillance system. Although COVID‐19 cases with mild symptoms are more likely to remain undetected, we expect the proportion of underdiagnosed severe COVID‐19 cases to be negligible.

Another limitation of our analysis is that we assumed instantaneous transitions through the analyzed pandemic phases, roughly corresponding to the times at which different variants became dominant (Table S1). Furthermore, during the COVID‐19 pandemic, social mixing patterns may have been altered by NPIs and behavioral changes in the population. We could not consider changes in the age‐specific proportion of contacts over time in absence of longitudinal data on contact patterns by age collected during the pandemic period. This is obviously a simplification as, for example, some restrictions targeted preferentially contacts in specific age groups (e.g., school closure). Despite these conservative assumptions, we show that the model approximates well age‐specific trends in SARS‐CoV‐2 infection dynamics (Figure S4) as well as observed temporal changes in seroprevalence (Figure 4).

Estimates of severity and lethality are essential to assess the true burden of COVID‐19 on health care systems and to evaluate the effectiveness and cost‐effectiveness of control interventions. They are often measured as the fraction of severe or lethal cases among the reported ones (e.g., case hospitalization ratio or case fatality ratio), as these values are easily obtainable from surveillance data. However, these measures may change depending on case‐finding efforts that are in place at a given time place. Less biased and more comparable measures of severity and lethality are IHR, IIR, and IFR. They are especially difficult to estimate because they require knowledge on the number of infections (a variable proportion of which goes undiagnosed). Here, we propose a method to estimate them from another quantity derived from surveillance data, namely the time‐varying reproduction number Rt.

Most studies estimating the proportions of severe outcomes among SARS‐CoV‐2 infections focus on COVID‐19 deaths and provide estimates of the IFR at specific time‐points, applying statistical regression models to serological data, possibly in association with contact tracing records.^46^, ^47^, ^48^ One previously published SARS‐CoV‐2 transmission model provides estimates of the IHR and IFR over time in United Kingdom using data from repeated serological surveys.^54^ The dependence of these approaches on serological data limits their applicability to settings where serological surveys were performed, and estimates obtained need to be interpreted considering the study population and the time point of data collection. One key advantage of our approach is that it relies on surveillance data routinely collected at the national level and can be applied in the absence of up‐to‐date serological data. Moreover, this study is among the first ones providing estimates of IHR, IIR, and IFR both in the pre‐ and post‐vaccination period.^50^, ^55^ The application of our methodology to assess changes in COVID‐19 severity in real‐time may be limited by the lack of up‐to‐date estimates on vaccine effectiveness and cross‐protection against circulating variants. However, we believe that our approach may be suitable to assess in quasi‐real‐time the severity and lethality of a newly emerging pathogen in absence of vaccination.

Quantitative estimates provided in this study apply to the case of Italy and may depend on the many country‐specific factors that characterized the response to the COVID‐19 pandemic, such as governmental choices on the adoption of NPIs, or differences in COVID‐19 vaccines uptake. Despite these heterogeneities, estimates of the Italian SARS‐CoV‐2 seroprevalence obtained through the proposed modeling approach are in line with those estimated for high‐income European countries.^35^, ^56^ We thus expect the general trends and conclusions of this study may apply also to other high‐income European countries, as well as to other countries that have adopted a similar mitigation approach throughout the pandemic. Finally, our results suggest that our approach may represent a valid alternative to assess SARS‐CoV‐2 infection rates based on routinely collected surveillance data, when serological data are not available.

Despite the large number of confirmed SARS‐CoV‐2 infections in 2022, also fueled by the emergence of new partially immune‐escaping Omicron sub‐variants (e.g., BA.2 and BA.5) and recombinant lineages, the burden of COVID‐19 in Italy had a manageable impact on hospitals. However, the possible future emergence of new variants that may escape previous immunity (natural or from vaccine) and are more transmissible and/or pathogenic stresses the need of maintaining careful genomic surveillance on SARS‐CoV‐2 variants and epidemic trends.^57^

## AUTHOR CONTRIBUTIONS

**Valentina Marziano**: Conceptualization (equal); data curation (supporting); formal analysis (supporting); investigation (lead); methodology (lead); software (lead); validation (equal); visualization (lead); writing—original draft (lead); writing—review & editing (lead). **Giorgio Guzzetta**: Investigation (equal); methodology (equal); funding acquisition (lead); project administration (lead); visualization (equal); writing—original draft (equal); writing—review & editing (equal). **Francesco Menegale**: Investigation (supporting); methodology (supporting); visualization (supporting); writing—review & editing (supporting). **Chiara Sacco**: Data curation (equal); formal analysis (equal); resources (equal); writing—review & editing (supporting). **Daniele Petrone**: Data curation (equal); formal analysis (equal); resources (equal); writing—review & editing (supporting). **Alberto Mateo Urdiales**: Data curation (equal); formal analysis (equal); resources (equal); writing—review & editing (supporting). **Martina Del Manso**: Data curation (equal); formal analysis (equal); resources (equal); writing—review & editing (supporting). **Antonino Bella**: Data curation (equal); formal analysis (equal); resources (equal); writing—review & editing (supporting). **Massimo Fabiani**: Data curation (equal); formal analysis (equal); resources (equal); writing—review & editing (supporting). **Maria Fenicia Vescio**: Data curation (equal); formal analysis (equal); resources (equal); writing—review & editing (supporting). **Flavia Riccardo**: Data curation (equal); formal analysis (equal); resources (equal); writing—review & editing (supporting). **Piero Poletti**: Investigation (supporting); methodology (supporting); supervision (supporting); visualization (supporting); writing—review & editing (equal). **Mattia Manica**: Investigation (supporting); methodology (supporting); visualization (supporting); writing—review & editing (supporting). **Agnese Zardini**: Investigation (supporting); methodology (supporting); visualization (supporting); writing—review & editing (supporting). **Valeria d'Andrea**: Investigation (supporting); methodology (supporting); visualization (supporting); writing—review & editing (supporting). **Filippo Trentini**: Investigation (supporting); methodology (supporting); visualization (supporting); writing—review & editing (supporting). **Paola Stefanelli**: Data curation (equal); formal analysis (equal); resources (equal); writing—review & editing (supporting). **Giovanni Rezza**: Data curation (equal); formal analysis (equal); resources (equal); writing—review & editing (supporting). **Anna Teresa Palamara**: Data curation (equal); formal analysis (equal); resources (lead); supervision (supporting); writing—review & editing (supporting). **Silvio Brusaferro**: Data curation (equal); formal analysis (equal); resources (lead); supervision (supporting); writing—review & editing (supporting). **Ajelli Marco**: Conceptualization (lead); investigation (equal); methodology (equal); supervision (equal); visualization (supporting); writing—review & editing (equal). **Patrizio Pezzotti**: Conceptualization (equal); data curation (lead); formal analysis (lead); resources (lead); supervision (equal); writing—review & editing (equal). **Stefano Merler**: Conceptualization (lead); funding acquisition (lead); investigation (supporting); methodology (supporting); project administration (lead); supervision (lead); writing—review & editing (equal).

## CONFLICT OF INTEREST STATEMENT

MA has received research funding from Seqirus. The funding is not related to COVID‐19. PS has received funding from GSK, not related to this project. All other authors declare no conflicts of interest.

### PEER REVIEW

The peer review history for this article is available at https://www.webofscience.com/api/gateway/wos/peer-review/10.1111/irv.13181.

## Supporting information


**Figure S1.** Schematic representation model transitions.
**Table S1.** Prevalence of SARS‐CoV‐2 variants as estimated from selected flash surveys conducted in Italy,^6^ with corresponding conventional date of transition to dominance assumed in this study.
**Figure S2.** Vaccination coverage by age group as observed in Italy between December 27, 2020, and February 20, 2022.^21^
**Table S2.** Description of key parameters and assumptions used in the main analysis. If not specified, values are set equal to those reported in the column “ancestral phases”.
**Table S3.** Number of SARS‐CoV‐2 confirmed infections as reported to the national integrated surveillance system during phase p and those who were admitted to the hospital, to the ICU, or died, respectively, during phase p.^29,34^
**Figure S3.** COVID‐19 deaths over the first ancestral phase (in thousands).
**Table S4.** Description and assumptions on the model parameters that are varied in the sensitivity analyses. Highlighted parameter values are those that are varied with respect to the main analysis.
**Figure S4.** a Age distribution of SARS‐CoV‐2 confirmed infections reported to the national integrated surveillance system in the ancestral phases (red), in the Alpha phase (orange), in the Delta phase (blue) and in the Omicron phase (green).^34^ b Mean age distribution of SARS‐CoV‐2 infections as estimated by the model in the different phases.
**Figure S5.** Changes in SARS‐CoV‐2 infection hospitalization ratio (IHR) by age group. Bars: mean estimates; vertical lines: 95% CI; n = 300 stochastic model realizations.
**Figure S6.** Changes in SARS‐CoV‐2 infection ICU ratio (IIR) by age group. Bars: mean estimates; vertical lines: 95% CI; n = 300 stochastic model realizations.
**Figure S7.** Changes in SARS‐CoV‐2 infection fatality ratio (IFR) by age group. Bars: mean estimates; vertical lines: 95% CI; n = 300 stochastic model realizations.
**Figure S8.** Grey bars represent the daily number of SARS‐CoV‐2 tests administered per 1000 individuals.^37^ Background colors indicate the classification in different phases, and the dates indicated within the graph denote the day of transition between consecutive phases. The vertical dotted line denotes the start of the vaccination campaign on December 27, 2020.
**Figure S9.** a Proportion of vaccinated individuals (one or more doses, independently from the time at which vaccination was administered) among SARS‐CoV‐2 confirmed infections reported to the national integrated surveillance system in the ancestral phases (red), in the Alpha phase (orange), in the Delta phase (blue) and in the Omicron phase (green).^34^ b Proportion of vaccinated individuals (one or more doses, independently from the time at which vaccination was administered) among SARS‐CoV‐2 infections estimated by the model in the different phases. Bars: mean estimates; vertical lines: 95% CI; n = 300 stochastic model realizations. c Proportion of the Italian population vaccinated with one or more doses at the end of the different phases.^21^
**Figure S10.** SARS‐CoV‐2 cumulative incidence and infection ascertainment ratio (IAR) as obtained assuming a shorter duration of immunity after natural infection (sensitivity A).
**Figure S11.** Changes in SARS‐CoV‐2 infection hospitalization ratio, ICU ratio and fatality ratio as obtained assuming a shorter duration of immunity after natural infection (sensitivity A).
**Figure S12.** SARS‐CoV‐2 cumulative incidence and infection ascertainment ratio (IAR) as obtained assuming a longer duration of immunity after natural infection (sensitivity B).
**Figure S13.** Changes in SARS‐CoV‐2 infection hospitalization ratio, ICU ratio and fatality ratio as obtained assuming a longer duration of immunity after natural infection (sensitivity B).
**Figure S14.** SARS‐CoV‐2 cumulative incidence and infection ascertainment ratio (IAR) as obtained assuming a shorter duration of vaccine protection (sensitivity C).
**Figure S15.** Changes in SARS‐CoV‐2 infection hospitalization ratio, ICU ratio and fatality ratio as obtained assuming a shorter duration of vaccine protection (sensitivity C).
**Figure S16.** SARS‐CoV‐2 cumulative incidence and infection ascertainment ratio (IAR) as obtained assuming a longer duration of vaccine protection (sensitivity D).
**Figure S17.** Changes in SARS‐CoV‐2 infection hospitalization ratio, ICU ratio and fatality ratio as obtained assuming a longer duration of vaccine protection (sensitivity D).
**Figure S18.** SARS‐CoV‐2 cumulative incidence and infection ascertainment ratio (IAR) as obtained assuming that infection with previous lineages grant a lower level of cross‐protection against re‐infection with Omicron BA.1 (sensitivity E).
**Figure S19.** Changes in SARS‐CoV‐2 infection hospitalization ratio, ICU ratio and fatality ratio as obtained assuming that infection with previous lineages grant a lower level of cross‐protection against re‐infection with Omicron BA.1 (sensitivity E).
**Figure S20.** SARS‐CoV‐2 cumulative incidence and infection ascertainment ratio (IAR) as obtained by assuming an age‐dependent susceptibility for SARS‐CoV‐2 variants (sensitivity F).
**Figure S21.** Changes in SARS‐CoV‐2 infection hospitalization ratio, ICU ratio and fatality ratio as obtained by assuming an age‐dependent susceptibility for SARS‐CoV‐2 variants (sensitivity F).

## Data Availability

Processed data and code used in this study are available on Zenodo at https://doi.org/10.5281/zenodo.8006661.

## References

[1] Koelle K , Martin MA , Antia R , Lopman B , Dean NE . The changing epidemiology of SARS‐CoV‐2. Science. 2022;375(6585):1116‐1121. doi:10.1126/science.abm4915 35271324 PMC9009722

[2] Guzzetta G , Riccardo F , Marziano V , et al. Impact of a Nationwide lockdown on SARS‐CoV‐2 transmissibility, Italy. Emerg Infect Dis. 2021;27(1):267‐270. doi:10.3201/eid2701.202114 33080168 PMC7774526

[3] Pan A , Liu L , Wang C , et al. Association of public health interventions with the epidemiology of the COVID‐19 outbreak in Wuhan, China. JAMA. 2020;323(19):1915‐1923. doi:10.1001/jama.2020.6130 32275295 PMC7149375

[4] Di Domenico L , Pullano G , Sabbatini CE , Boëlle PY , Colizza V . Impact of lockdown on COVID‐19 epidemic in Île‐de‐France and possible exit strategies. BMC Med. 2020;18(1):240. doi:10.1186/s12916-020-01698-4 32727547 PMC7391016

[5] Coletti P , Libin P , Petrof O , et al. A data‐driven metapopulation model for the Belgian COVID‐19 epidemic: assessing the impact of lockdown and exit strategies. BMC Infect Dis. 2021;21(1):503. doi:10.1186/s12879-021-06092-w 34053446 PMC8164894

[6] Marziano V , Guzzetta G , Rondinone BM , et al. Retrospective analysis of the Italian exit strategy from COVID‐19 lockdown. Proc Natl Acad Sci. 2021;118(4):e2019617118. doi:10.1073/pnas.2019617118 33414277 PMC7848712

[7] Mathieu E , Ritchie H , Ortiz‐Ospina E , et al. A global database of COVID‐19 vaccinations. Nat Hum Behav. 2021;5(7):947‐953. doi:10.1038/s41562-021-01122-8 33972767

[8] Stefanelli P , Trentini F , Guzzetta G , et al. Co‐circulation of SARS‐CoV‐2 alpha and gamma variants in Italy, February and march 2021. Eurosurveillance. 2022;27(5):2100429. doi:10.2807/1560-7917.ES.2022.27.5.2100429 35115077 PMC8815098

[9] Davies NG , Abbott S , Barnard RC , et al. Estimated transmissibility and impact of SARS‐CoV‐2 lineage B.1.1.7 in England. Science. 2021;372(6538):eabg3055. doi:10.1126/science.abg3055 33658326 PMC8128288

[10] Alizon S , Haim‐Boukobza S , Foulongne V , et al. Rapid spread of the SARS‐CoV‐2 Delta variant in some French regions, June 2021. Eurosurveillance. 2021;26(28):2100573. doi:10.2807/1560-7917.ES.2021.26.28.2100573 34269174 PMC8284044

[11] Ferguson NM . B.1.617.2 transmission in England: risk factors and transmission advantage. Published online June 2021:14.

[12] Stefanelli P , Trentini F , Petrone D , et al. Tracking the progressive spread of the SARS‐CoV‐2 omicron variant in Italy, December 2021 to January 2022. Eurosurveillance. 2022;27(45):2200125. doi:10.2807/1560-7917.ES.2022.27.45.2200125 36367013 PMC9650705

[13] Task force COVID‐19 del Dipartimento Malattie Infettive, Servizio di Informatica, Istituto Superiore di Sanità . Epidemia COVID‐19. Aggiornamento Nazionale: 2 Marzo 2022. https://www.epicentro.iss.it/coronavirus/bollettino/Bollettino-sorveglianza-integrata-COVID-19_2-marzo-2022.pdf

[14] Covid‐19 Opendata Vaccini. https://github.com/italia/covid19-opendata-vaccini

[15] Riccardo F , Ajelli M , Andrianou XD , et al. Epidemiological characteristics of COVID‐19 cases and estimates of the reproductive numbers 1 month into the epidemic, Italy, 28 January to 31 March 2020. Eurosurveillance. 2020;25(49):2000790. doi:10.2807/1560-7917.ES.2020.25.49.2000790 33303064 PMC7730489

[16] Yang J , Marziano V , Deng X , et al. Despite vaccination, China needs non‐pharmaceutical interventions to prevent widespread outbreaks of COVID‐19 in 2021. Nat Hum Behav. 2021;5(8):1009‐1020. doi:10.1038/s41562-021-01155-z 34158650 PMC8373613

[17] Marziano V , Guzzetta G , Mammone A , et al. The effect of COVID‐19 vaccination in Italy and perspectives for living with the virus. Nat Commun. 2021;12(1):7272. doi:10.1038/s41467-021-27532-w 34907206 PMC8671442

[18] Istituto Superiore di Sanità . COVID‐19 ISS Open Data – EpiCentro. https://www.epicentro.iss.it/coronavirus/open-data/covid_19-iss.xlsx

[19] Istituto Superiore di Sanità . Monitoraggio delle varianti del virus SARS‐CoV‐2 di interesse in sanità pubblica in Italia. https://www.epicentro.iss.it/coronavirus/sars-cov-2-monitoraggio-varianti-indagini-rapide

[20] Mossong J , Hens N , Jit M , et al. Social contacts and mixing patterns relevant to the spread of infectious diseases. PLoS Med. 2008;5(3):e74. doi:10.1371/journal.pmed.0050074 18366252 PMC2270306

[21] Diekmann O , Heesterbeek JAP , Metz JAJ . On the definition and the computation of the basic reproduction ratio R0 in models for infectious diseases in heterogeneous populations. J Math Biol. 1990;28(4):365‐382. doi:10.1007/BF00178324 2117040

[22] Diekmann O , Heesterbeek J , Roberts MG . The construction of next‐generation matrices for compartmental epidemic models. J R Soc Interface. 2010;7(47):873‐885. doi:10.1098/rsif.2009.0386 19892718 PMC2871801

[23] Cori A , Ferguson NM , Fraser C , Cauchemez S . A new framework and software to estimate time‐varying reproduction numbers during epidemics. Am J Epidemiol. 2013;178(9):1505‐1512. doi:10.1093/aje/kwt133 24043437 PMC3816335

[24] Thompson RN , Stockwin JE , van Gaalen RD , et al. Improved inference of time‐varying reproduction numbers during infectious disease outbreaks. Epidemics. 2019;29:100356. doi:10.1016/j.epidem.2019.100356 31624039 PMC7105007

[25] Fabiani M , Puopolo M , Filia A , et al. Effectiveness of an mRNA vaccine booster dose against SARS‐CoV‐2 infection and severe COVID‐19 in persons aged ≥60 years and other high‐risk groups during predominant circulation of the delta variant in Italy, 19 July to 12 December 2021. Expert Rev Vaccines. 2022;21(7):975‐982. doi:10.1080/14760584.2022.2064280 35389748 PMC9115794

[26] Fabiani M , Puopolo M , Morciano C , et al. Effectiveness of mRNA vaccines and waning of protection against SARS‐CoV‐2 infection and severe covid‐19 during predominant circulation of the delta variant in Italy: retrospective cohort study. BMJ. 2022;376:e069052. doi:10.1136/bmj-2021-069052 35144968 PMC8829820

[27] Andrews N , Stowe J , Kirsebom F , et al. Covid‐19 vaccine effectiveness against the omicron (B.1.1.529) variant. N Engl J Med. 2022;386(16):1532‐1546. doi:10.1056/NEJMoa2119451 35249272 PMC8908811

[28] Harris RJ , Hall JA , Zaidi A , Andrews NJ , Dunbar JK , Dabrera G . Effect of vaccination on household transmission of SARS‐CoV‐2 in England. N Engl J Med. 2021;385(8):759‐760. doi:10.1056/NEJMc2107717 34161702 PMC8262621

[29] Lipsitch M , Kahn R . Interpreting vaccine efficacy trial results for infection and transmission. Vaccine. 2021;39(30):4082‐4088. doi:10.1016/j.vaccine.2021.06.011 34130883 PMC8197448

[30] Altarawneh HN , Chemaitelly H , Hasan MR , et al. Protection against the omicron variant from previous SARS‐CoV‐2 infection. N Engl J Med. 2022;386(13):1288‐1290. doi:10.1056/NEJMc2200133 35139269 PMC8849180

[31] Andeweg SP , de Gier B , Eggink D , et al. Protection of COVID‐19 vaccination and previous infection against omicron BA.1, BA.2 and Delta SARS‐CoV‐2 infections. Nat Commun. 2022;13(1):4738. doi:10.1038/s41467-022-31838-8 35961956 PMC9373894

[32] Hall VJ , Foulkes S , Charlett A , et al. SARS‐CoV‐2 infection rates of antibody‐positive compared with antibody‐negative health‐care workers in England: a large, multicentre, prospective cohort study (SIREN). The Lancet. 2021;397(10283):1459‐1469. doi:10.1016/S0140-6736(21)00675-9 PMC8040523 33844963

[33] Andrews N , Tessier E , Stowe J , et al. Duration of protection against mild and severe disease by Covid‐19 vaccines. N Engl J Med. 2022;386(4):340‐350. doi:10.1056/NEJMoa2115481 35021002 PMC8781262

[34] Menegale F , Manica M , Zardini A , et al. Evaluation of waning of SARS‐CoV‐2 vaccine‐induced immunity: a systematic review and meta‐analysis. JAMA Netw Open. 2023;6(5):e2310650. doi:10.1001/jamanetworkopen.2023.10650 37133863 PMC10157431

[35] Bergeri I , Whelan MG , Ware H , et al. Global SARS‐CoV‐2 seroprevalence from January 2020 to April 2022: a systematic review and meta‐analysis of standardized population‐based studies. PLoS Med. 2022;19(11):e1004107. doi:10.1371/journal.pmed.1004107 36355774 PMC9648705

[36] Poletti P , Tirani M , Cereda D , et al. Association of age with likelihood of developing symptoms and critical disease among close contacts exposed to patients with confirmed SARS‐CoV‐2 infection in Italy. JAMA Netw Open. 2021;4(3):e211085. doi:10.1001/jamanetworkopen.2021.1085 33688964 PMC7948061

[37] Thomas SJ , Moreira ED , Kitchin N , et al. Safety and efficacy of the BNT162b2 mRNA Covid‐19 vaccine through 6 months. N Engl J Med. 2021;385(19):1761‐1773. doi:10.1056/NEJMoa2110345 34525277 PMC8461570

[38] El Sahly HM , Baden LR , Essink B , et al. Efficacy of the mRNA‐1273 SARS‐CoV‐2 vaccine at completion of blinded phase. N Engl J Med. 2021;385(19):1774‐1785. doi:10.1056/NEJMoa2113017 34551225 PMC8482810

[39] Joung SY , Ebinger JE , Sun N , et al. Awareness of SARS‐CoV‐2 omicron variant infection among adults with recent COVID‐19 seropositivity. JAMA Netw Open. 2022;5(8):e2227241. doi:10.1001/jamanetworkopen.2022.27241 35976645 PMC9386542

[40] Petherick A , Goldszmidt R , Andrade EB , et al. A worldwide assessment of changes in adherence to COVID‐19 protective behaviours and hypothesized pandemic fatigue. Nat Hum Behav. 2021;5(9):1145‐1160. doi:10.1038/s41562-021-01181-x 34345009

[41] Campbell F , Archer B , Laurenson‐Schafer H , et al. Increased transmissibility and global spread of SARS‐CoV‐2 variants of concern as at June 2021. Eurosurveillance. 2021;26(24):2100509. doi:10.2807/1560-7917.ES.2021.26.24.2100509 34142653 PMC8212592

[42] Sacco C , Petrone D , Manso MD , et al. Risk and protective factors for SARS‐CoV‐2 reinfections, surveillance data, Italy, August 2021 to March 2022. Eurosurveillance. 2022;27(20):2200372. doi:10.2807/1560-7917.ES.2022.27.20.2200372 35593164 PMC9121659

[43] Bager P , Wohlfahrt J , Bhatt S , et al. Risk of hospitalisation associated with infection with SARS‐CoV‐2 Omicron variant versus Delta variant in Denmark: an observational cohort study. Lancet Infect Dis. 2022;22(7):967‐976. doi:10.1016/S1473-3099(22)00154-2 35468331 PMC9033212

[44] Wolter N , Jassat W , Walaza S , et al. Early assessment of the clinical severity of the SARS‐CoV‐2 omicron variant in South Africa: a data linkage study. The Lancet. 2022;399(10323):437‐446. doi:10.1016/S0140-6736(22)00017-4 PMC8769664 35065011

[45] Salje H , Tran Kiem C , Lefrancq N , et al. Estimating the burden of SARS‐CoV‐2 in France. Science. 2020;369(6500):208‐211. doi:10.1126/science.abc3517 32404476 PMC7223792

[46] Zardini A , Galli M , Tirani M , et al. A quantitative assessment of epidemiological parameters required to investigate COVID‐19 burden. Epidemics. 2021;37:100530. doi:10.1016/j.epidem.2021.100530 34826786 PMC8595250

[47] Poletti P , Tirani M , Cereda D , et al. Age‐specific SARS‐CoV‐2 infection fatality ratio and associated risk factors, Italy, February to April 2020. Eurosurveillance. 2020;25(31):2001383. doi:10.2807/1560-7917.ES.2020.25.31.2001383 32762797 PMC7459272

[48] COVID‐19 Forecasting Team . Variation in the COVID‐19 infection–fatality ratio by age, time, and geography during the pre‐vaccine era: a systematic analysis. The Lancet. 2022;399(10334):1469‐1488. doi:10.1016/S0140-6736(21)02867-1 PMC8871594 35219376

[49] Khandaker G , Dierig A , Rashid H , King C , Heron L , Booy R . Systematic review of clinical and epidemiological features of the pandemic influenza A (H1N1) 2009. Influenza Other Respi Viruses. 2011;5(3):148‐156. doi:10.1111/j.1750-2659.2011.00199.x PMC5657010 21477133

[50] Ferrante P . The first 2 years of COVID‐19 in Italy: incidence, lethality, and health policies. Front Public Health. 2022;10. Accessed June 1, 2023:986743. doi:10.3389/fpubh.2022.986743 36388357 PMC9664068

[51] Asch DA , Sheils NE , Islam MN , et al. Variation in US hospital mortality rates for patients admitted with COVID‐19 during the first 6 months of the pandemic. JAMA Intern Med. 2021;181(4):471‐478. doi:10.1001/jamainternmed.2020.8193 33351068 PMC7756246

[52] Trentini F , Marziano V , Guzzetta G , et al. Pressure on the health‐care system and intensive care utilization during the COVID‐19 outbreak in the Lombardy region of Italy: a retrospective observational study in 43,538 hospitalized patients. Am J Epidemiol. 2022;191(1):137‐146. doi:10.1093/aje/kwab252 34652416 PMC8549288

[53] Sacco C , Mateo‐Urdiales A , Petrone D , et al. Estimating averted COVID‐19 cases, hospitalisations, intensive care unit admissions and deaths by COVID‐19 vaccination, Italy, January−September 2021. Eurosurveillance. 2021;26(47):2101001. doi:10.2807/1560-7917.ES.2021.26.47.2101001 34823637 PMC8619872

[54] Knock ES , Whittles LK , Lees JA , et al. Key epidemiological drivers and impact of interventions in the 2020 SARS‐CoV‐2 epidemic in England. Sci Transl Med. 2021;13(602):eabg4262. doi:10.1126/scitranslmed.abg4262 34158411 PMC8432953

[55] Eales O , Haw D , Wang H , et al. Dynamics of SARS‐CoV‐2 infection hospitalisation and infection fatality ratios over 23 months in England. PLoS Biol. 2023;21(5):e3002118. doi:10.1371/journal.pbio.3002118 37228015 PMC10212114

[56] Groenheit R , Bacchus P , Galanis I , et al. High prevalence of SARS‐CoV‐2 Omicron infection despite high seroprevalence, Sweden, 2022. Emerg Infect Dis. 2023;29(6):1240‐1243. doi:10.3201/eid2906.221862 37141616 PMC10202879

[57] Markov PV , Katzourakis A , Stilianakis NI . Antigenic evolution will lead to new SARS‐CoV‐2 variants with unpredictable severity. Nat Rev Microbiol. 2022;20(5):251‐252. doi:10.1038/s41579-022-00722-z 35288685 PMC8919145

