# Supplementary material for: Estimating SARS‐CoV‐2 infections and associated changes in COVID‐19 severity and fatality
Source: Influenza Other Respir Viruses. 2023 Aug 16;17(8):e13181. doi: 10.1111/irv.13181 (PMC10432583; doi:10.1111/irv.13181)
Supplement: Supplementary file 1 — Figure S1. Schematic representation model transitions. Table S1. Prevalence of SARS‐CoV‐2 variants as estimated from selected flash surveys conducted in Italy,6 with corresponding conventional date of transition to dominance assumed in this study. Figure S2. Vaccination coverage by age group as observed in Italy between December 27, 2020, and February 20, 2022.21 Table S2. Description of key parameters and assumptions used in the main analysis. If not specified, values are set equal to those reported in the column “ancestral phases”. Table S3. Number of SARS‐CoV‐2 confirmed infections as reported to the national integrated surveillance system during phase p and those who were admitted to the hospital, to the ICU, or died, respectively, during phase p.29,34 Figure S3. COVID‐19 deaths over the first ancestral phase (in thousands). Table S4. Description and assumptions on the model parameters that are varied in the sensitivity analyses. Highlighted parameter values are those that are varied with respect to the main analysis. Figure S4. a Age distribution of SARS‐CoV‐2 confirmed infections reported to the national integrated surveillance system in the ancestral phases (red), in the Alpha phase (orange), in the Delta phase (blue) and in the Omicron phase (green).34 b Mean age distribution of SARS‐CoV‐2 infections as estimated by the model in the different phases. Figure S5. Changes in SARS‐CoV‐2 infection hospitalization ratio (IHR) by age group. Bars: mean estimates; vertical lines: 95% CI; n = 300 stochastic model realizations. Figure S6. Changes in SARS‐CoV‐2 infection ICU ratio (IIR) by age group. Bars: mean estimates; vertical lines: 95% CI; n = 300 stochastic model realizations. Figure S7. Changes in SARS‐CoV‐2 infection fatality ratio (IFR) by age group. Bars: mean estimates; vertical lines: 95% CI; n = 300 stochastic model realizations. Figure S8. Grey bars represent the daily number of SARS‐CoV‐2 tests administered per 1000 individuals.37 Background colors indic [file IRV-17-e13181-s001.docx]

**Supporting Information**

**Estimating SARS-CoV-2 infections and associated changes in COVID-19 severity and fatality**

Valentina Marziano ^1^, Giorgio Guzzetta ^1^, Francesco Menegale ^1,2^, Chiara Sacco ^3^, Daniele Petrone ^3^, Alberto Mateo Urdiales ^3^, Martina Del Manso ^3^, Antonino Bella ^3^, Massimo Fabiani ^3^, Maria Fenicia Vescio ^3^, Flavia Riccardo ^3^, Piero Poletti ^1^, Mattia Manica ^1^, Agnese Zardini ^1^, Valeria d’Andrea ^1^, Filippo Trentini ^1,4,5^, Paola Stefanelli ^3^, Giovanni Rezza ^6^, Anna Teresa Palamara ^3^, Silvio Brusaferro ^3^, Marco Ajelli ^7,#^, Patrizio Pezzotti ^3,#^, Stefano Merler ^1,#,*^

^1^ Center for Health Emergencies, Bruno Kessler Foundation, Trento, Italy

^2^ Department of Mathematics, University of Trento, Trento, Italy

^3^ Department of Infectious Diseases, Istituto Superiore di Sanità, Rome, Italy

^4^ Dondena Centre for Research on Social Dynamics and Public Policy, Bocconi University, Milan, Italy

^5^ Covid Crisis Lab, Bocconi University, Italy

^6^ Health Prevention directorate, Ministry of Health, Rome, Italy

^7^ Laboratory for Computational Epidemiology and Public Health, Department of Epidemiology and Biostatistics, Indiana University School of Public Health, Bloomington, IN, USA

^#^ Senior authors

^*^ Corresponding author: [merler@fbk.eu](mailto:merler@fbk.eu)

Contents

[1. Materials and Methods 2](#_Toc137805793)

[1.1 Model for SARS-CoV-2 transmission and vaccination 2](#_Toc137805794)

[1.2 Reproducing the COVID-19 epidemic trajectory in Italy 10](#_Toc137805795)

[1.3 Model outputs 11](#_Toc137805796)

[1.4 Model initialization 12](#_Toc137805797)

[1.5 Sensitivity analyses 13](#_Toc137805798)

[2. Additional results 15](#_Toc137805799)

[2.1 Main analysis 15](#_Toc137805800)

[2.2 Sensitivity analyses 18](#_Toc137805801)

[Duration of immunity after natural infection 18](#_Toc137805802)

[Duration of protection from vaccination 20](#_Toc137805803)

[Cross-protection against Omicron provided by infection with previous variants 22](#_Toc137805804)

[Susceptibility to infection for SARS-CoV-2 variants 23](#_Toc137805805)

[References 25](#_Toc137805806)

# 1. Materials and Methods

## 1.1 Model for SARS-CoV-2 transmission and vaccination

We developed an age-structured stochastic model for SARS-CoV-2 transmission and vaccination, based on a susceptible-infectious-removed-susceptible scheme (SIRS) and adapted from previously published models ^1,2^

The model was used to simulate the evolution of COVID-19 epidemiology in Italy between February 21, 2020, and February 20, 2022.

The simulation period is conventionally subdivided in the following phases:

“ancestral (phase 1)”, from February 21 to June 30, 2020. This period was characterized by a first pandemic wave and the circulation of SARS-CoV-2 ancestral lineages. During this phase the Italian health care system was put under substantial pressure ^3^ and the spread of SARS-CoV-2 was contained through a strict national lockdown ^4^.

“ancestral (phase 2)”, from July 1, 2020, to February 17, 2021. This second phase was characterized by a summer period of low incidence and by a second pandemic wave in the fall of 2020, still caused by ancestral lineages. The second pandemic wave was countered through the introduction of a tier-based reactive restriction system ^5^. On December 27, 2020, the vaccination campaign was launched.

“Alpha (phase 3)”, from February 18, 2021, to July 1, 2021. This phase was characterized by the dominant circulation of the Alpha variant ^6^ (Table S1) and by a substantial scale-up in COVID-19 vaccination rates (Figure 1b in the main text).

“Delta (phase 4)”, from July 2, 2021, to December 23, 2021. This phase was characterized by the dominant circulation of the Delta variant ^6^ (Table S1) and by the progression of the vaccination campaign, including the administration of booster doses.

“Omicron (phase 5)”, from December 24, 2021 to February 20, 2022, characterized by the dominant circulation of the Omicron BA.1 variant ^6^ (Table S1).

Mixing patterns were assumed to be heterogeneous across ages according to an age-specific social contact matrix estimated prior to the COVID-19 pandemic ^7^. We assumed an age-dependent susceptibility to SARS-CoV-2 infection in the ancestral phases: lower in children under 15 years of age and higher for the elderly (65+), compared to individuals of working age ^8^. For the Alpha, Delta, and Omicron phases susceptibility to SARS-CoV-2 infection was assumed homogeneous across ages.

We simulate a vaccination campaign including the administration of first, second doses and booster doses. Vaccination is assumed to reduce the individuals’ susceptibility to SARS-CoV-2 infection. Breakthrough infections (i.e., infections in vaccinated individuals) are assumed to be half as infectious as those in unvaccinated individuals ^9,10^.

The model accounts for waning of both protection from natural infection and vaccination. Protection from natural immune response after infection with all lineages is assumed to wane exponentially with a constant rate over the considered period ^11^. Before waning, natural infection provides complete protection against re-infection with homologous and previous variants, while we assume a partial cross-protection against Omicron BA.1 infection ^12^ granted by natural infection with previous variants. Homologous re-infection without waning of natural immunity is not considered for any lineage. Ancestral lineages were replaced by the Alpha variant a few weeks after the start of the vaccination campaign in Italy and waning of vaccine protection estimated in the literature during dominance of the Alpha variant is negligible ^13,14^. Thus, we only considered waning of protection from vaccine-induced immune response after two doses of vaccine and booster in the Delta and Omicron phases ^14–16^. Before waning of vaccine protection, vaccination reduces the probability of infection (with different efficacy estimates for the different endpoints and considered strains). After waning of immunity either from natural infection or vaccination, individuals are considered susceptible to infection with the same risk of unvaccinated individuals who were never exposed to SARS-CoV-2.

**Figure S1. Schematic representation model transitions.** Blue compartments represent unvaccinated individuals and therefore eligible for vaccination with the first dose; green compartments represent vaccinated individuals. **a** ancestral, Alpha and Delta phases**. b** Omicron phase.

Model transitions are summarized in the schematic representations in Figure S1 and described in detail by the following system of differential equations:

$$\left\{ \begin{aligned} S_{a}^{'}\left( t \right)=-\lambda_{S,a}\left( t \right)S_{a}\left( t \right){-\alpha}_{a}\left( t \right)S_{a}\left( t \right)+\upsilon_{R}\left[ R_{\text{ancestral},a}\left( t \right)+R_{\text{alpha},a}\left( t \right)+R_{\text{delta},a}\left( t \right) \right] \\ I_{a}^{'}(t)= \lambda_{S,a}\left( t \right)S_{a}\left( t \right)-\gamma I_{a}\left( t \right)+f_{\text{omicron}}\left( t \right)\lambda_{R,a}\left( t \right)[R_{\text{ancestral},a}\left( t \right)+R_{\text{alpha},a}\left( t \right)+R_{\text{delta},a}\left( t \right)] \\ R_{\text{ancestral},a}^{'}\left( t \right)= f_{\text{ancestral}}\left( t \right)\gamma I_{a}\left( t \right)-\alpha_{a}\left( t \right)R_{\text{ancestral},a}\left( t \right)-\upsilon_{R}R_{\text{ancestral},a}\left( t \right)-f_{\text{omicron}}\left( t \right)\lambda_{R,a}\left( t \right)R_{\text{ancestral},a}\left( t \right) \\ R_{\text{alpha},a}^{'}\left( t \right)= f_{\text{alpha}}\left( t \right)\gamma I_{a}\left( t \right)-\alpha_{a}\left( t \right)R_{\text{alpha},a}\left( t \right)-\upsilon_{R}R_{\text{alpha},a}\left( t \right)-f_{\text{omicron}}\left( t \right)\lambda_{R,a}\left( t \right)R_{\text{alpha},a}\left( t \right) \\ R_{\text{delta},a}^{'}\left( t \right)= f_{\text{delta}}\left( t \right)\gamma I_{a}\left( t \right)-\alpha_{a}\left( t \right)R_{\text{delta},a}\left( t \right)-\upsilon_{R}R_{\text{delta},a}\left( t \right)-f_{\text{omicron}}\left( t \right)\lambda_{R,a}\left( t \right)R_{\text{delta},a}\left( t \right) \\ R_{\text{omicron},a}^{'}\left( t \right)= f_{\text{omicron}}\left( t \right)\gamma I_{a}\left( t \right)-\alpha_{a}\left( t \right)R_{\text{omicron},a}\left( t \right) \\ V_{1,a}^{'}\left( t \right)=\alpha_{a}\left( t \right)S_{a}\left( t \right)-\lambda_{V_{1},a}\left( t \right)V_{1,a}(t)-{\omega_{1}V}_{1,a}\left( t \right) \\ V_{2,a}^{'}\left( t \right)={\omega_{1}V}_{1,a}\left( t \right)-\lambda_{V_{2},a}\left( t \right)V_{2,a}(t)-{\omega_{2}V}_{2,a}\left( t \right) \\ V_{3,a}^{'}\left( t \right)={\omega_{2}V}_{2,a}\left( t \right)-\lambda_{V_{3},a}\left( t \right)V_{3,a}(t)-{\omega_{3}V}_{3,a}\left( t \right) \\ V_{4,a}^{'}\left( t \right)={\omega_{3}V}_{3,a}\left( t \right)-\lambda_{V_{4},a}\left( t \right)V_{4,a}(t)-\upsilon_{V}(t)V_{4,a}(t)-\beta_{a}\left( t \right)V_{4,a}(t) \\ V_{5,a}^{'}\left( t \right)=\upsilon_{V}(t)V_{4,a}(t)-\lambda_{V_{5},a}\left( t \right)V_{5,a}(t)-\beta_{a}\left( t \right)V_{5,a}(t) \\ B_{1,a}^{'}\left( t \right)=\beta_{a}\left( t \right)\left[ V_{4,a}\left( t \right)+V_{5,a}\left( t \right) \right]-\lambda_{B_{1},a}\left( t \right)B_{1,a}\left( t \right)- \upsilon_{B}(t)B_{1,a}\left( t \right) \\ B_{2,a}^{'}\left( t \right)=\upsilon_{B}(t)B_{1,a}\left( t \right)-\lambda_{B_{2},a}\left( t \right)B_{2,a}\left( t \right) \\ U_{1,\text{ancestral},a}^{'}\left( t \right)= \alpha_{a}\left( t \right)R_{\text{ancestral},a}\left( t \right)-\left[ \omega_{1}\omega_{2}/(\omega_{1}+\omega_{2}) \right]U_{\text{1,ancestral},a}\left( t \right)-f_{\text{omicron}}\left( t \right)\lambda_{U_{1},a}\left( t \right)U_{\text{1,ancestral},a}\left( t \right) \\ U_{2,\text{ancestral},a}^{'}\left( t \right)= \left[ \omega_{1}\omega_{2}/(\omega_{1}+\omega_{2}) \right]U_{\text{1,ancestral},a}\left( t \right)-f_{\text{omicron}}\left( t \right)\lambda_{U_{2},a}\left( t \right)U_{\text{2,ancestral},a}\left( t \right) \\ U_{\text{1,alpha},a}^{'}\left( t \right)= \alpha_{a}\left( t \right)R_{\text{alpha},a}\left( t \right)-\left[ \omega_{1}\omega_{2}/(\omega_{1}+\omega_{2}) \right]U_{\text{1,alpha},a}\left( t \right)-f_{\text{omicron}}\left( t \right)\lambda_{U_{1},a}\left( t \right)U_{\text{1,alpha},a}\left( t \right) \\ U_{2,\text{alpha},a}^{'}\left( t \right)= \left[ \omega_{1}\omega_{2}/(\omega_{1}+\omega_{2}) \right]U_{\text{1,alpha},a}\left( t \right)-f_{\text{omicron}}\left( t \right)\lambda_{U_{2},a}\left( t \right)U_{\text{2,alpha},a}\left( t \right) \\ U_{1,\text{delta},a}^{'}\left( t \right)= \alpha_{a}\left( t \right)R_{\text{delta},a}\left( t \right)-\left[ \omega_{1}\omega_{2}/(\omega_{1}+\omega_{2}) \right]U_{\text{1},\text{delta},a}\left( t \right)-f_{\text{omicron}}\left( t \right)\lambda_{U_{1},a}\left( t \right)U_{\text{1,delta},a}\left( t \right) \\ U_{2,\text{delta},a}^{'}\left( t \right)= \left[ \omega_{1}\omega_{2}/(\omega_{1}+\omega_{2}) \right]U_{\text{1,delta,}a}\left( t \right)-f_{\text{omicron}}\left( t \right)\lambda_{U_{2},a}\left( t \right)U_{\text{2,delta},a}\left( t \right) \\ U_{1,\text{omicron},a}^{'}\left( t \right)= \alpha_{a}\left( t \right)R_{\text{omicron},a}\left( t \right)-\left[ \omega_{1}\omega_{2}/(\omega_{1}+\omega_{2}) \right]U_{\text{1,omicron},a}\left( t \right) \\ U_{2,\text{omicron},a}^{'}\left( t \right)= \left[ \omega_{1}\omega_{2}/(\omega_{1}+\omega_{2}) \right]U_{\text{1,omicron},a}\left( t \right) \\ {I_{a}^{V}}^{'}(t)= \sum_{k=1}^{5} [\lambda_{V_{k},a}\left( t \right)V_{k,a}(t)]+\sum_{k=1}^{2} [\lambda_{B_{k},a}\left( t \right)B_{k,a}(t)]+ \\ +f_{\text{omicron}}\left( t \right)\sum_{k=1}^{2} \left[ \lambda_{U_{k},a}\left( t \right)\left( U_{k\text{,ancestral},a}\left( t \right)+U_{k\text{,alpha},a}\left( t \right)+U_{k\text{,delta},a}\left( t \right) \right) \right]+ \\ +f_{\text{omicron}}\left( t \right)\lambda_{R^{V},a}\left( t \right)\left[ R_{\text{ancestral},a}^{V}\left( t \right)+R_{\text{alpha},a}^{V}\left( t \right)+R_{\text{delta},a}^{V}\left( t \right) \right]-\gamma I_{a}^{V}\left( t \right) \\ {R_{\text{ancestral},a}^{V}}^{'}\left( t \right)=f_{\text{ancestral}}\left( t \right)\gamma I_{a}^{V}\left( t \right)-f_{\text{omicron}}\left( t \right)\lambda_{R^{V},a}\left( t \right)R_{\text{ancestral},a}^{V}\left( t \right) \\ {R_{\text{alpha},a}^{V}}^{'}\left( t \right)=f_{\text{alpha}}\left( t \right)\gamma I_{a}^{V}\left( t \right)-f_{\text{omicron}}\left( t \right)\lambda_{R^{V},a}\left( t \right)R_{\text{alpha},a}^{V}\left( t \right) \\ {R_{\text{delta},a}^{V}}^{'}\left( t \right)=f_{\text{delta}}\left( t \right)\gamma I_{a}^{V}\left( t \right)-f_{\text{omicron}}\left( t \right)\lambda_{R^{V},a}\left( t \right)R_{\text{delta},a}^{V}\left( t \right) \\ {R_{\text{omicron},a}^{V}}^{'}\left( t \right)=f_{\text{omicron}}(t)\gamma I_{a}^{V}\left( t \right) \end{aligned} \right.$$

where:

the functions $f_{\mathrm{ancestral}}$; $f_{\mathrm{alpha}}$; $f_{\mathrm{delta}}$ and $f_{\mathrm{omicron}}$are step functions defined as follows:

$$f_{\mathrm{ancestral}}\left( t \right)=\left\{ \begin{aligned} 1, &T_{\text{0}}\leq t<T_{\text{alpha}} \\ 0, &\text{otherwise} \end{aligned} \right.$$

$$f_{\mathrm{alpha}}\left( t \right)=\left\{ \begin{aligned} 1, &T_{\text{alpha}}\leq t<T_{\text{delta}} \\ 0, &\text{otherwise} \end{aligned} \right.$$

$$f_{\mathrm{delta}}\left( t \right)=\left\{ \begin{aligned} 1, &T_{\text{delta}}\leq t<T_{\text{omicron}} \\ 0, &\text{otherwise} \end{aligned} \right.$$

$$f_{\mathrm{omicron}}\left( t \right)=\left\{ \begin{aligned} 1, &T_{\text{omicron}}\leq t\leq T_{\text{max}} \\ 0, &\text{otherwise} \end{aligned} \right.$$

where $T_{\text{0}}$ is the day of simulation start (February 21, 2020); $T_{\text{alpha}}$ is the first day of the Alpha phase (February 18, 2021); $T_{\text{delta}}$ is the first day of the Delta phase (July 2, 2021); $T_{\text{omicron}}$ is the first day of the Omicron phase (December 24, 2021) and $T_{\text{max}}$ is the last day of simulations (February 20, 2022). These dates of transition were conventionally defined based on when variants were assumed to become dominant (prevalence >50%), taking reference from genomic surveillance estimates of the prevalence of SARS-CoV-2 lineages ^6^, reported in in Table S1. The date of transition between the first and the second ancestral phases will be denoted with $T_{\text{ancestral (phase 2)}}$ and was conventionally defined as July 1^st^, 2020. By February 20, 2022, the last day of simulations, Omicron BA.1 was still prevalent in Italy.

**Table S1**. Prevalence of SARS-CoV-2 variants as estimated from selected flash surveys conducted in Italy ^6^, with corresponding conventional date of transition to dominance assumed in this study.

| **SARS-CoV-2 variant** | **Last estimate before dominance** | | **First estimate after dominance** | | **Date of transition** |
| --- | --- | --- | --- | --- | --- |
|  | **Date** | **Prevalence** | **Date** | **Prevalence** |  |
| Alpha | Feb 5, 2021 | 17.8% | Feb 18, 2021 | 54.0% | Feb 18, 2021 ($T_{\text{alpha}}$) |
| Delta | Jun 22, 2021 | 22.7% | Jul 20, 2021 | 94.8% | Jul 2, 2021 ($T_{\text{delta}}$) |
| Omicron BA.1 | Dec 20, 2021 | 21.0% | Jan 3, 2022 | 80.8% | Dec 24, 2021 ($T_{\text{omicron}}$) |

$S_{a}(t)$ represents the number of unvaccinated individuals in the age group $a$ who are unprotected against SARS-CoV-2 infection at time $t$.

$I_{a}(t)$represents the number of infectious unvaccinated individuals in the age group $a$ at time $t$. Infectious individuals are assumed to have been infected with ancestral lineages if $t<T_{\text{alpha}}$, with the Alpha variant if $T_{\text{alpha}}\leq t<T_{\text{delta}}$, with Delta if $T_{\text{delta}}\leq t<T_{\text{omicron}}$; and with Omicron BA.1 if $T_{\text{omicron}}\leq t\leq T_{\text{max}}$.

$R_{\text{ancestral},a}\left( t \right);R_{\text{alpha},a}\left( t \right); R_{\text{delta},a}\left( t \right); R_{\text{omicron},a}(t)$represent the number of unvaccinated individuals in the age group $a$ who at time $t$ have recovered from infection with the different strains and for whom natural immunity has not waned.

$V_{1,a}(t);V_{2,a}(t);V_{3,a}(t);V_{4,a}(t)$; $V_{5,a}(t)$; $B_{1,a}(t)$ and $B_{2,a}(t)$ represent the number of vaccinated individuals at different stages of protection at time $t$. In particular,

$V_{1,a}$denotes individuals in the age group $a$ vaccinated with the first dose, for whom the first dose is not effective yet.

$V_{2,a}$denotes individuals in the age group $a$ vaccinated with the first dose, for whom the first dose is effective.

$V_{3,a}$ denotes individuals in the age group $a$ vaccinated with the second dose for whom the second dose is not effective yet.

$V_{4,a}$denotes individuals in the age group $a$ vaccinated with the second dose for whom the second dose is effective.

$V_{5,a}$denotes individuals in the age group $a$ vaccinated with the second dose for whom vaccine protection has waned.

$B_{1,a}$denotes individuals in the age group $a$ vaccinated with the booster dose for whom the booster dose is effective.

$B_{2,a}$denotes individuals in the age group $a$ vaccinated with the booster dose for whom vaccine protection has waned.

Compartments $V_{1,a}$ and $V_{3,a}$encode the delay required for the mounting of an effective immune response.

$U_{\text{1,ancestral},a}(t); U_{\text{1,alpha},a}(t); U_{\text{1,delta},a}(t);U_{\text{1,omicron},a}(t)$represent the number of individuals in the age group $a$ who at time $t$ have been vaccinated with the first dose, despite having already experienced SARS-CoV-2 infection with the corresponding strains, and for whom the first dose is effective.

$U_{\text{2,ancestral},a}(t); U_{\text{2,alpha},a}(t); U_{\text{2,delta},a}(t);U_{\text{2,omicron},a}(t)$represent the number of individuals in the age group $a$ who at time $t$ are vaccinated with the second dose despite having already experienced SARS-CoV-2 infection with each of the considered strains, and for whom the second dose is effective.

$I_{a}^{V}(t)$represents the number of infectious individuals in the age group $a$ at time $t$ among those who have already received at least one vaccine dose. Infectious individuals are assumed to have been infected with ancestral lineages if $t<T_{\text{alpha}}$, with the Alpha variant if $T_{\text{alpha}}\leq t<T_{\text{delta}}$, with Delta if $T_{\text{delta}}\leq t<T_{\text{omicron}}$ and with Omicron BA.1 if $T_{\text{omicron}}\leq t\leq T_{\text{max}}$.

$R_{\text{ancestral},a}^{V}(t); R_{\text{alpha},a}^{V}(t); R_{\text{delta},a}^{V}(t); R_{\text{omicron},a}^{V}(t)$ represent the number of individuals in the age group $a$ who at time $t$ have recovered from natural infection with the different strains, contracted after having received at least one vaccine dose.

The model described by the system of ordinary differential equations above is implemented through a stochastic discrete-time model with a time step $\tau=0.25$ days.

**Force of infection**

Unvaccinated individuals who are unprotected against SARS-CoV-2 infection ($S$) are exposed to a time and age-dependent force of infection$\lambda_{S,a}\left( t \right)$ which is defined as:

$$\lambda_{S,a}\left( t \right)=\delta\left( t \right)r_{a}(t)\sum_{\tilde{a}} C_{a,\tilde{a}}\frac{I_{\tilde{a}} \left( t \right)+\pi I_{\tilde{a}}^{V}\left( t \right)}{N_{\tilde{a}}}$$

where:

$\delta\left( t \right)$ is a multiplicative factor shaping SARS-CoV-2 transmissibility at time $t$ (see Section 1.2 for details);

$r_{a}(t)$is the relative susceptibility to SARS-CoV-2 infection at age $a$. For ancestral phases (${t<T}_{\text{alpha}}$), we sample susceptibility profiles from the posterior distribution estimated in ^8^, having mean values $r_{a}(t)$*=*0.58 (95%CI 0.34-0.98) under 15 years of age*;* $r_{a}(t)$=1 between 15 and 64 years; and $r_{a}(t)$*=*1.65 (95%CI 1.03-2.65) above 64 years. From the Alpha phase on ($t\geq$ $T_{\mathrm{alpha}}$), we assume $r_{a}(t)$=1 for all ages.

$C_{a,\tilde{a}}$ represents the age-group-specific contact matrix, as estimated before the SARS-CoV-2 pandemic ^7^, whose entries describe the mean numbers of persons in age group $\tilde{a}$ encountered by an individual of age group $a$ in an average day.

$\pi$ represents the relative infectiousness of SARS-CoV-2 infections among vaccinated compared to unvaccinated. We assumed $\pi=0.5$ ^9,10^.

$N_{\tilde{a}}$ represents the number of individuals in the age group $\tilde{a}$.

Vaccinated individuals can get infected with any SARS-CoV-2 variant with a susceptibility that depends on vaccination stage and the effectiveness of the vaccine against the circulating variant. Individuals who have recovered from natural infection with ancestral lineages, Alpha or Delta variant may get re-infected with Omicron BA.1 due to its ability to escape immunity from natural infection with heterologous lineages ^12^.

We modeled the age-dependent force of infection$\lambda_{D,a}\left( t \right)$ for individuals in compartment $D$ as follows:

$$\lambda_{D,a}\left( t \right)=(1-\chi_{D}(t))\lambda_{S,a}\left( t \right)$$

where$\chi_{D}$(t) is a time-dependent scaling factor accounting for the reduction in susceptibility of compartment $D$ compared to unprotected unvaccinated individuals ($S$) at time $t$. For vaccinated compartments, the scaling factor $\chi_{D}$(t) represents the effectiveness of vaccines in preventing infection; for unvaccinated compartments recovered from natural infection with ancestral lineages, Alpha or Delta variants ($R_{\text{ancestral}}$; $R_{\text{alpha}}$ and $R_{\text{delta}}$), $\chi_{D}$(t) represents the effectiveness of infection with previous lineages in preventing reinfection with Omicron BA.1. Values assumed for $\chi_{D}(t)$ are reported in Table S2.

Protection from natural immune response in unvaccinated individuals recovered from SARS-CoV-2 infection is assumed to wane exponentially with a constant rate $\upsilon_{R}$=1/730 days^-1^ ^11^. In our model, we assume that the duration of infectiousness is exponentially distributed with mean $1/\gamma$ and that infectiousness is constant over time. Under these assumptions, the generation time of the model is also exponentially distributed with the same mean value as the infectious period ^17^. To obtain a mean generation time of 6.6 days within the model, as estimated in the literature for SARS-CoV-2 ancestral lineages in Italy ^18^, we set the value of the parameter $\gamma$=0.1515152 for all infectious compartments. The value of $\gamma$ is kept constant across different phases, based on published estimates of a substantially unchanged value of the generation time in Italy between ancestral lineages, Alpha, Delta and Omicron BA.1 ^18–20^.

**Allocation of vaccine doses**

The great majority of vaccines administered in Italy during the period of interest were two-dose vaccines: BNT162b2 (65% of doses), mRNA-1273 (25% of doses), and ChAdOx1 nCoV-19 (9% of doses), thus, we simulate a two-dose vaccination campaign and a booster campaign ^21^. The rollout of vaccination is modeled using detailed data on the daily age-specific number of first and booster doses administered over the considered period ^21^. The priority order of the Italian vaccination campaign was based on the WHO SAGE roadmap ^22^ prioritizing high-risk population age segments, i.e. over 80 years of age and essential workers (e.g. health care workers and teachers) and then progressively targeting younger age groups. The same priority order was also followed during rollout of the booster vaccination campaign, started in autumn 2021.

In Italy, infectious individuals were not considered eligible for vaccination during the COVID-19 vaccination campaign. Accordingly, in the model, we assume that the first dose of vaccination is administered to unvaccinated individuals who are unprotected against SARS-CoV-2 infection or have recovered from natural infection with all considered strains, while we exclude currently infectious individuals.

At each time $t$, the number of unvaccinated individuals in the age group $a$ who will receive a first vaccine dose is determined as a fraction $z_{a}\left( t \right)$ of the corresponding population:

$$z_{a}\left( t \right)=\frac{d_{a}(t)}{S_{a}\left( t \right)+ R_{a}\left( t \right)}$$

where $d_{a}(t)$ represents the number of first vaccine doses administered to individuals in the age group $a$ at time $t$; and ${R_{a}\left( t \right)=R}_{\text{ancestral,}a}+$ $R_{\text{alpha,}a}+ R_{\text{delta,}a}+R_{\text{omicron,}a}$. The value of $d_{a}(t)$ is inferred from data on the daily number of first doses administered by age group in Italy between the start of vaccination (December 27, 2020) and the end of the simulated period (February 20, 2022) ^21^.

The first-dose vaccination rate $\alpha_{a}\left( t \right)$ associated to the probability $z_{a}\left( t \right)$ in the differential equations model can be computed trough the following equation:

$z_{a}\left( t \right)=1-e^{-\alpha_{a}\left( t \right)\tau}$.

where $\tau=0.25$days is the time step of our simulations.

We assume that the first dose becomes effective on average after ${1/\omega}_{1}=14$ days from its administration ^16,23^. The second dose is administered on average 28 days after the first dose (i.e. ${1/\omega}_{1}+{1/\omega}_{2}=\frac{\omega_{1}{+\omega}_{2}}{\omega_{1}\omega_{2}}=28$ days and ${1/\omega}_{2}= 14$days) ^24^ to all individuals who have received one dose of vaccine and did not contract the infection while waiting for the second one. We assume that the second dose becomes effective after ${1/\omega}_{3}=14$ days from its administration ^16,23^.

Booster doses are administered to individuals who have received two doses of vaccination. At each time *t*, the number of individuals in the age group $a$ who will receive a booster vaccine dose is determined as a fraction $w_{a}\left( t \right)$ of the corresponding population:

$$w_{a}\left( t \right)=\frac{b_{a}(t)}{V_{4,a}\left( t \right)+V_{5,a}\left( t \right)+U_{2,a}\left( t \right)+R_{a}^{V}\left( t \right)}$$

where $b_{a}(t)$ represents the number of booster doses administered to individuals in the age group $a$ at time $t$; $U_{2,a}\left( t \right)=U_{2,\text{ancestral,}a}\left( t \right)+U_{2,\text{alpha,}a}\left( t \right)+U_{2,\text{delta,}a}\left( t \right)+U_{2,\text{omicron,}a}\left( t \right)$ and $R_{a}^{V}\left( t \right)=R_{\text{ancestral,}a}^{V}\left( t \right)+R_{\text{alpha,}a}^{V}\left( t \right)+R_{\text{delta,}a}^{V}\left( t \right)+R_{\text{omicron,}a}^{V}\left( t \right).$ The value of $b_{a}(t)$ is inferred from data on the daily number of booster doses administered by age group in Italy in the simulation period ^21^.

The booster-dose vaccination rate $\beta_{a}\left( t \right)$ associated to the probability $w_{a}\left( t \right)$ in the differential equations model can be computed through the following equation:

$w_{a}\left( t \right)=1-e^{-\beta_{a}\left( t \right)\tau}$.

First-dose vaccination and booster vaccination coverage by age over the simulated period are shown in Figure S2.


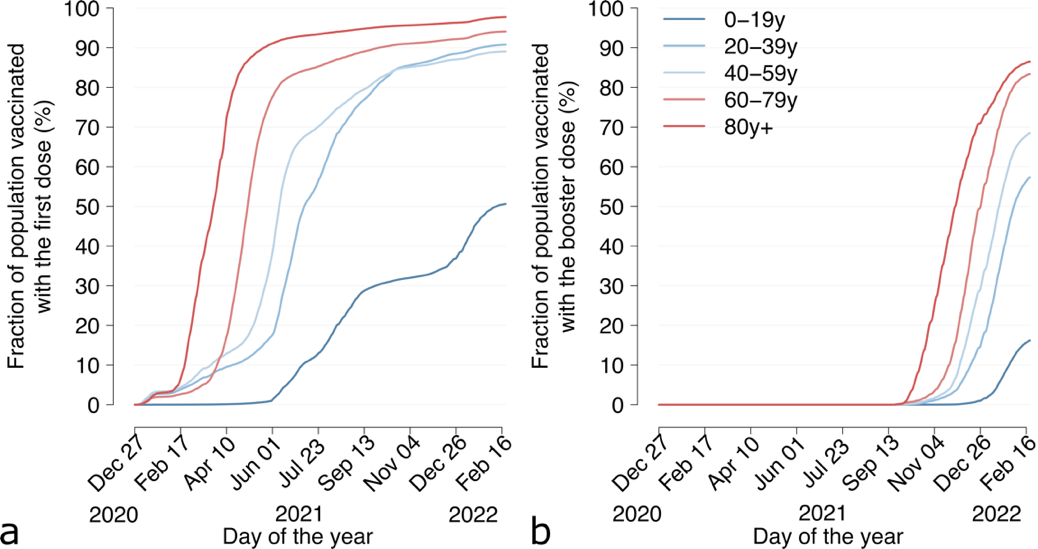


**Figure S2. Vaccination coverage by age group as observed in Italy between December 27, 2020, and February 20, 2022** ^21^**. a** first dose; **b** booster dose. In Italy, administration of two doses is recommended to all individuals aged 5 years or more; administration of a booster dose is recommended to all individuals aged 12 years or more.

We consider waning of protection after two doses of vaccine and after the booster dose ^25^. Average durations of protection after two doses and after one booster dose considered for different variants $p$ are reported in Table S2. Due to the different waning rates of vaccine protection across variants, the proportion of vaccine-protected individuals changes when a new variant becomes dominant. For example, the proportion of vaccinees with booster of age $a$ that were protected against Omicron BA.1 infection at the time of its dominance $T_{\text{omicron}}$is approximated by

$P_{a}=\int_{0}^{T_{\text{omicron}}} b_{a}\left( t \right)e^{-(T_{\text{omicron}}-t)\upsilon_{B,\text{omicron}}}dt$.

Since there were

$$Q_{a}=\int_{0}^{T_{\text{omicron}}-1} b_{a}\left( t \right)e^{-(T_{\text{omicron}}-1-t)\upsilon_{B,\text{delta}}}dt$$

individuals of age $a$ that were protected against Delta infection at date $T_{\text{omicron}}-1$, on day $T_{\text{omicron}}$we move $Q_{a}-P_{a}$ individuals from compartment $B_{1,a}$ (booster protected) to compartment $B_{2,a}$ (booster waned). We perform the same operation for all variants and for the two-dose compartment as well.

**Table S2**. Description of key parameters and assumptions used in the main analysis. If not specified, values are set equal to those reported in the column “ancestral phases”.

| **Parameter** | **Ancestral, phases 1 and 2**  ($t<T_{\text{alpha}}$) | **Alpha, phase 3**  ($T_{\text{alpha}}\leq t<T_{\text{delta}}$) | **Delta, phase 4**  ($T_{\text{delta}}\leq t<T_{\text{omicron}}$) | **Omicron, phase 5**  ($t\geq T_{\text{omicron}}$) | **Source** |  |
| --- | --- | --- | --- | --- | --- | --- |
| **Epidemiological** | | | | | |  |
| Generation time (1/$\gamma)$ | 6.6 days | - | - | - | ^18^ |  |
| Susceptibility to infection ($r_{a}(t)$) | Age-dependent  - $r_{a}\left( t \right)$=0.58 (95%CI 0.34-0.98) under 15 years;  - $r_{a}(t)$=1 between 15 and 64 years;  - $r_{a}(t)$=1.65 (95%CI 1.03-2.65) over 64 years | Homogenous:  $r_{a}(t)$=1 for all ages | Homogenous:  $r_{a}(t)$=1 for all ages | Homogenous:  $r_{a}(t)$=1 for all ages | ^8^ |  |
| Average duration of immunity after natural infection ($1/\upsilon_{R}$) | 2 years | - | - | - | ^11^ |  |
| Age-group specific contact matrix ($C_{a,\tilde{a}}$*)* | Contact matrix estimated for Italy before the pandemic | - | - | - | ^7^ |  |
| **Vaccination** | | | | | |  |
| Delay between 1^st^ dose and achievement of vaccine efficacy ($1/\omega_{1}$) | 14 days | - | - | - | ^14^ |  |
| Interval between 1^st^ and 2^nd^ dose (1/$\omega_{1}+$1/$\omega_{2}$) | 28 days | - | - | - | ^24^ |  |
| Delay between 2^nd^ dose and achievement of vaccine efficacy ($1/\omega_{3}$) | 14 days | - | - | - | ^14^ |  |
| Duration of vaccine protection after two doses of vaccine ($1/\upsilon_{V}(t)$) | no waning  ($\upsilon_{V}\left( t \right)=\upsilon_{V,\text{ancestral}}=0$) | no waning ($\upsilon_{V}\left( t \right)=\upsilon_{V,\text{alpha}}=0$) | $1/\upsilon_{V,\text{delta}}=$200.6 days | $1/\upsilon_{V,\text{omicron}}=$74.5 days | ^25^ |  |
| Duration of vaccine protection after booster ($1/\upsilon_{B}(t)$) | no waning  ($\upsilon_{B}\left( t \right)=\upsilon_{B,\text{ancestral}}=0$) | no waning  ($\upsilon_{B}\left( t \right)=\upsilon_{B,\text{alpha}}=0$) | no waning  ($\upsilon_{B}\left( t \right)=\upsilon_{B,\text{delta}}=0$) | $1/\upsilon_{B,\text{omicron}}=$ 195.3 days | ^25^ |  |
| Relative infectiousness of SARS-CoV-2 breakthrough infections ($\pi$) | 50% | - | - | - | ^9,10^ |  |
| **Susceptibility reduction in compartment D compared to unvaccinated individuals who are unprotected against SARS-CoV-2** ($\chi_{D}\left( t \right)$) | | | | | | |
| $\chi_{V_{1}}(t)=\chi_{V_{5}}(t)= \chi_{B_{2}}(t)$ | 0 | 0 | 0 | 0 | Assumed |  |
| $\chi_{V_{2}}\left( t \right)=$ $\chi_{V_{3}}(t)$ | 0.49* | 0.49 | 0.502 | 0.428 | ^14,15^ |  |
| $\chi_{V_{4}}(t)$ | 0.79* | 0.79 | 0.69 | 0.655 | ^14,15^ |  |
| $\chi_{B_{1}}(t)$ | 0 | 0 | 0.761 | 0.669 | ^15,16^ |  |
| $\chi_{U_{1,\text{ancestral}}}\left( t \right)=\chi_{U_{1,\text{alpha}}}\left( t \right)=\chi_{U_{1,\text{delta}}}\left( t \right)=$  $\chi_{U_{2,\text{ancestral}}}\left( t \right)=\chi_{U_{2,\text{alpha}}}\left( t \right)=\chi_{U_{2,\text{delta}}}\left( t \right)=$  $\chi_{R_{\text{ancestral}}^{V}}\left( t \right)=\chi_{R_{\text{alpha}}^{V}}\left( t \right)=\chi_{R_{\text{delta}}^{V}}\left( t \right)$ | 1 | 1 | 1 | 0.669** | Assumed |  |
| $\chi_{R_{\text{ancestral}}}\left( t \right)=\chi_{R_{\text{alpha}}}\left( t \right)=\chi_{R_{\text{delta}}}\left( t \right)$ | 1 | 1 | 1 | 0.56 | ^12^ |  |

*assumed equal to estimates available for the Alpha phase **assumed equal to estimates of protection from booster in the Omicron phase

## 1.2 Reproducing the COVID-19 epidemic trajectory in Italy

The reproduction number associated to the dynamical system above can be computed as the dominant eigenvalue of the Next Generation Matrix (NGM) ^26–28^, defined as:

$NGM=\frac{\delta\left( t \right)}{\gamma}\left( \begin{matrix} B_{a,\tilde{a}}^{S,i_{S}} & B_{a,\tilde{a}}^{S,i_{V_{1}}} & B_{a,\tilde{a}}^{S,i_{V_{2}}} & B_{a,\tilde{a}}^{S,i_{V_{3}}} & B_{a,\tilde{a}}^{S,i_{V_{4}}} & B_{a,\tilde{a}}^{S,i_{V_{5}}} & B_{a,\tilde{a}}^{S,i_{B_{1}}} & B_{a,\tilde{a}}^{S,i_{B_{2}}} & B_{a,\tilde{a}}^{S,i_{U_{1}}} & B_{a,\tilde{a}}^{S,i_{U_{2}}} & B_{a,\tilde{a}}^{S,i_{R_{V}}} & B_{a,\tilde{a}}^{S,i_{R}} \\ B_{a,\tilde{a}}^{V_{1},i_{S}} & B_{a,\tilde{a}}^{V_{1},i_{V_{1}}} & B_{a,\tilde{a}}^{V_{1},i_{V_{2}}} & B_{a,\tilde{a}}^{V_{1},i_{V_{3}}} & B_{a,\tilde{a}}^{V_{1},i_{V_{4}}} & B_{a,\tilde{a}}^{V_{1},i_{V_{5}}} & B_{a,\tilde{a}}^{V_{1},i_{B_{1}}} & B_{a,\tilde{a}}^{V_{1},i_{B_{2}}} & B_{a,\tilde{a}}^{V_{1},i_{U_{1}}} & B_{a,\tilde{a}}^{V_{1},i_{U_{2}}} & B_{a,\tilde{a}}^{V_{1},i_{R_{V}}} & B_{a,\tilde{a}}^{V_{1},i_{R}} \\ B_{a,\tilde{a}}^{V_{2},i_{S}} & B_{a,\tilde{a}}^{V_{2},i_{V_{1}}} & B_{a,\tilde{a}}^{V_{2},i_{V_{2}}} & B_{a,\tilde{a}}^{V_{2},i_{V_{3}}} & B_{a,\tilde{a}}^{V_{2},i_{V_{4}}} & B_{a,\tilde{a}}^{V_{2},i_{V_{5}}} & B_{a,\tilde{a}}^{V_{2}i_{B_{1}}} & B_{a,\tilde{a}}^{V_{2},i_{B_{2}}} & B_{a,\tilde{a}}^{V_{2},i_{U_{1}}} & B_{a,\tilde{a}}^{V_{2},i_{U_{2}}} & B_{a,\tilde{a}}^{V_{2},i_{R_{V}}} & B_{a,\tilde{a}}^{V_{2},i_{R}} \\ B_{a,\tilde{a}}^{V_{3},i_{S}} & B_{a,\tilde{a}}^{V_{3},i_{V_{1}}} & B_{a,\tilde{a}}^{V_{3},i_{V_{2}}} & B_{a,\tilde{a}}^{V_{3},i_{V_{3}}} & B_{a,\tilde{a}}^{V_{3},i_{V_{4}}} & B_{a,\tilde{a}}^{V_{3},i_{V_{5}}} & B_{a,\tilde{a}}^{V_{3},i_{B_{1}}} & B_{a,\tilde{a}}^{V_{3},i_{B_{2}}} & B_{a,\tilde{a}}^{V_{3},i_{U_{1}}} & B_{a,\tilde{a}}^{V_{3},i_{U_{2}}} & B_{a,\tilde{a}}^{V_{3},i_{R_{V}}} & B_{a,\tilde{a}}^{V_{3},i_{R}} \\ B_{a,\tilde{a}}^{V_{4},i_{S}} & B_{a,\tilde{a}}^{V_{4},i_{V_{1}}} & B_{a,\tilde{a}}^{V_{4},i_{V_{2}}} & B_{a,\tilde{a}}^{V_{4},i_{V_{3}}} & B_{a,\tilde{a}}^{V_{4},i_{V_{4}}} & B_{a,\tilde{a}}^{V_{4},i_{V_{5}}} & B_{a,\tilde{a}}^{V_{4},i_{B_{1}}} & B_{a,\tilde{a}}^{V_{4},i_{B_{2}}} & B_{a,\tilde{a}}^{V_{4},i_{U_{1}}} & B_{a,\tilde{a}}^{V_{4},i_{U_{2}}} & B_{a,\tilde{a}}^{V_{4},i_{R_{V}}} & B_{a,\tilde{a}}^{V_{4},i_{R}} \\ B_{a,\tilde{a}}^{V_{5},i_{S}} & B_{a,\tilde{a}}^{V_{5},i_{V_{1}}} & B_{a,\tilde{a}}^{V_{5},i_{V_{2}}} & B_{a,\tilde{a}}^{V_{5},i_{V_{3}}} & B_{a,\tilde{a}}^{V_{5},i_{V_{4}}} & B_{a,\tilde{a}}^{V_{5},i_{V_{5}}} & B_{a,\tilde{a}}^{V_{5},i_{B_{1}}} & B_{a,\tilde{a}}^{V_{5},i_{B_{2}}} & B_{a,\tilde{a}}^{V_{5,}i_{U_{1}}} & B_{a,\tilde{a}}^{V_{5},i_{U_{2}}} & B_{a,\tilde{a}}^{V_{5},i_{R_{V}}} & B_{a,\tilde{a}}^{V_{5},i_{R}} \\ B_{a,\tilde{a}}^{B_{1},i_{S}} & B_{a,\tilde{a}}^{B_{1},i_{V_{1}}} & B_{a,\tilde{a}}^{B_{1},i_{V_{2}}} & B_{a,\tilde{a}}^{B_{1},i_{V_{3}}} & B_{a,\tilde{a}}^{B_{1},i_{V_{4}}} & B_{a,\tilde{a}}^{B_{1},i_{V_{5}}} & B_{a,\tilde{a}}^{B_{1},i_{B_{1}}} & B_{a,\tilde{a}}^{B_{1},i_{B_{2}}} & B_{a,\tilde{a}}^{B_{1},i_{U_{1}}} & B_{a,\tilde{a}}^{B_{1},i_{U_{2}}} & B_{a,\tilde{a}}^{B_{1},i_{R_{V}}} & B_{a,\tilde{a}}^{B_{1},i_{R}} \\ B_{a,\tilde{a}}^{B_{2},i_{S}} & B_{a,\tilde{a}}^{B_{2},i_{V_{1}}} & B_{a,\tilde{a}}^{B_{2},i_{V_{2}}} & B_{a,\tilde{a}}^{B_{2},i_{V_{3}}} & B_{a,\tilde{a}}^{B_{2},i_{V_{4}}} & B_{a,\tilde{a}}^{B_{2},i_{V_{5}}} & B_{a,\tilde{a}}^{B_{2},i_{B_{1}}} & B_{a,\tilde{a}}^{B_{2},i_{B_{2}}} & B_{a,\tilde{a}}^{B_{2},i_{U_{1}}} & B_{a,\tilde{a}}^{B_{2},i_{U_{2}}} & B_{a,\tilde{a}}^{B_{2},i_{R_{V}}} & B_{a,\tilde{a}}^{B_{2},i_{R}} \\ B_{a,\tilde{a}}^{U_{1},i_{S}} & B_{a,\tilde{a}}^{U_{1},i_{V_{1}}} & B_{a,\tilde{a}}^{U_{1},i_{V_{2}}} & B_{a,\tilde{a}}^{U_{1},i_{V_{3}}} & B_{a,\tilde{a}}^{U_{1},i_{V_{4}}} & B_{a,\tilde{a}}^{U_{1},i_{V_{5}}} & B_{a,\tilde{a}}^{U_{1},i_{B_{1}}} & B_{a,\tilde{a}}^{U_{1},i_{B_{2}}} & B_{a,\tilde{a}}^{U_{1},i_{U_{1}}} & B_{a,\tilde{a}}^{U_{1},i_{U_{2}}} & B_{a,\tilde{a}}^{U_{1},i_{R_{V}}} & B_{a,\tilde{a}}^{U_{1},i_{R}} \\ B_{a,\tilde{a}}^{U_{2},i_{S}} & B_{a,\tilde{a}}^{U_{2},i_{V_{1}}} & B_{a,\tilde{a}}^{U_{2},i_{V_{2}}} & B_{a,\tilde{a}}^{U_{2},i_{V_{3}}} & B_{a,\tilde{a}}^{U_{2},i_{V_{4}}} & B_{a,\tilde{a}}^{U_{2},i_{V_{5}}} & B_{a,\tilde{a}}^{U_{2},i_{B_{1}}} & B_{a,\tilde{a}}^{U_{2},i_{B_{2}}} & B_{a,\tilde{a}}^{U_{2},i_{U_{1}}} & B_{a,\tilde{a}}^{U_{2},i_{U_{2}}} & B_{a,\tilde{a}}^{U_{2},i_{R_{V}}} & B_{a,\tilde{a}}^{U_{2},i_{R}} \\ B_{a,\tilde{a}}^{R_{V},i_{S}} & B_{a,\tilde{a}}^{U_{2},i_{V_{1}}} & B_{a,\tilde{a}}^{R_{V},i_{V_{2}}} & B_{a,\tilde{a}}^{R_{V},i_{V_{3}}} & B_{a,\tilde{a}}^{R_{V},i_{V_{4}}} & B_{a,\tilde{a}}^{R_{V},i_{V_{5}}} & B_{a,\tilde{a}}^{R_{V},i_{B_{1}}} & B_{a,\tilde{a}}^{R_{V},i_{B_{2}}} & B_{a,\tilde{a}}^{R_{V},i_{U_{1}}} & B_{a,\tilde{a}}^{R_{V},i_{U_{2}}} & B_{a,\tilde{a}}^{R_{V},i_{R_{V}}} & B_{a,\tilde{a}}^{R_{V},i_{R}} \\ B_{a,\tilde{a}}^{R,i_{S}} & B_{a,\tilde{a}}^{R,i_{V_{1}}} & B_{a,\tilde{a}}^{R,i_{V_{2}}} & B_{a,\tilde{a}}^{R,i_{V_{3}}} & B_{a,\tilde{a}}^{R,i_{V_{4}}} & B_{a,\tilde{a}}^{R,i_{V_{5}}} & B_{a,\tilde{a}}^{R,i_{B_{1}}} & B_{a,\tilde{a}}^{R,i_{B_{2}}} & B_{a,\tilde{a}}^{R,i_{U_{1}}} & B_{a,\tilde{a}}^{R,i_{U_{2}}} & B_{a,\tilde{a}}^{R,i_{R_{V}}} & B_{a,\tilde{a}}^{R,i_{R}} \end{matrix} \right)$ (1)

Each block $B_{a,\tilde{a}}^{D,i_{D}}$describes the time-dependent contribution to the transmission of age-specific interactions between susceptible individuals in compartment $D$ and infectious individuals who were infected while being in compartment $D$ (here denoted as $i_{D}$).

Specifically, the explicit computation of the NGM starting from model equations yields:

$$B_{a,\tilde{a}}^{D,i_{D}}\left( t \right)=r_{a}(t)C_{a,\tilde{a}}\left[ 1-\chi_{D}(t) \right] \Theta_{i_{D}}\frac{N_{\tilde{a}}^{D}(t)}{N_{\tilde{a}}}$$

where:

$r_{a}(t)$is the relative susceptibility to SARS-CoV-2 infection at age $a$;

$C_{a,\tilde{a}}$ is the age-group-specific contact matrix ^7^,

$\chi_{D}(t)$ is the reduction in susceptibility to SARS-CoV-2 of compartment $D$ compared to unprotected unvaccinated individuals $S$ and $\chi_{S}(t)$is set to 0 by definition;

$\Theta_{i_{D}}$ is the relative infectiousness of SARS-CoV-2 infections among vaccinated compared to unvaccinated (equal to $\pi$ for vaccinated compartments, and 1 for unvaccinated compartments);

$N_{\tilde{a}}^{D}(t)$ is the number of individuals of age$\tilde{a}$ in compartment $D$ at time $t$;

$N_{\tilde{a}}$ represents the total population of age $\tilde{a}$*.*

To reproduce the epidemic trajectory observed in Italy over the study period we use Equation 1 to recalibrate daily the value of $\delta\left( t \right)$, given the distribution of the susceptibility profile by age ($r_{a}(t)$), the distribution of the bootstrapped contact matrix ($C_{a,\tilde{a}}$), and the values of $\gamma$, $\chi_{D}(t)$ and $\pi$ (see Table S2). For each day $t$, the selected value of $\delta\left( t \right)$ will be the one that will make the model’s reproduction number (recomputed after updating the $N_{\tilde{a}}^{D}(t)$ to current state variables of the model) match the corresponding value of the time-varying reproduction number Rt as estimated on the same day from epidemic curves collected by the national integrated surveillance system ^29–32^. Estimates of the time-varying reproduction number Rt were progressively published in the official weekly reports on COVID-19 in Italy (e.g., ^30^), the complete time-series of Rt used in this study can be found in the associated online repository ^33^. Results discussed in the main text and in the following sections were obtained by running 300 simulations, sampling at each run a different value from the joint distribution of the bootstrapped contact matrices $C_{a,\tilde{a}}$ and the relative susceptibility by age $r_{a}(t)$.

## 1.3 Model outputs

The main model output is the age-specific number of new infections per day $y_{a}^{D}\left( t \right)$ among individuals of age group $a$ in compartment $D$. Model outcomes are used, along with data on reported SARS-CoV-2 infections, COVID-19 hospitalizations, ICU admissions, and deaths ^29,34^ to provide estimates of the SARS-CoV-2 infection ascertainment ratio (IAR), infection hospitalization ratio (IHR), infection ICU ratio (IIR) and infection fatality ratio (IFR) in the different phases. For each model estimate, we report the mean values and 95% confidence intervals across stochastic simulations.

**SARS-CoV-2 infection rates and infection ascertainment ratio**

We computed the cumulative number of SARS-CoV-2 infections in the age group $a$ in the different phases as:

$$y_{a,\text{ancestral (phase 1)}}=\sum_{t=T_{0}}^{T_{\text{ancestral (phase 2)}}-1} \sum_{D} y_{a}^{D}\left( t \right)$$

$$y_{a,\text{ancestral (phase 2)}}=\sum_{t=T_{\text{ancestral (phase 2)}}}^{T_{\text{alpha}}-1} \sum_{D} y_{a}^{D}\left( t \right)$$

$$y_{a,\text{alpha}}=\sum_{t=T_{\text{alpha}}}^{T_{\text{delta}}-1} \sum_{D} y_{a}^{D}\left( t \right)$$

$$y_{a,\text{delta}}=\sum_{t=T_{\text{delta}}}^{T_{\text{omicron}}-1} \sum_{D} y_{a}^{D}\left( t \right)$$

$$y_{a,\text{omicron}}=\sum_{t=T_{\text{omicron}}}^{T_{\text{max}}} \sum_{D} y_{a}^{D}\left( t \right)$$

The age-specific SARS-CoV-2 cumulative incidence in the phase $p$ was then computed as $G_{a,p}=y_{a,p}/\text{pop}_{a}$, where $\text{pop}_{a}$ is the Italian population in age group $a$ ^35^.

The overall SARS-CoV-2 cumulative incidence in phase $p$ was computed as the ratio between the total number of SARS-CoV-2 infections estimated by the model in the same phase, $Y_{p}$, and the total Italian population:

$$G_{tot,p}=\frac{Y_{p}}{\sum_{a} \mathrm{pop}_{a}}=\frac{\sum_{a} y_{a,p}}{\sum_{a} \mathrm{pop}_{a}}$$

The infection ascertainment ratio $\mathrm{IAR}_{\text{p}}$ in phase $p$ was computed as the ratio between the number of infections reported to the national integrated surveillance system with a date of diagnosis in phase $p$($x_{\text{INF},p}$in Table S3) and the total number of SARS-CoV-2 infections estimated by the model in the same phase, $Y_{p}$.

**SARS-CoV-2 Infection hospitalization ratio, infection ICU ratio and infection fatality ratio**

For each phase $p$, we computed the infection hospitalization ratio ($\mathrm{IHR}_{p}$), infection ICU ratio ($\mathrm{IIR}_{p}$) and infection fatality ratio ($\mathrm{IFR}_{p}$) as follows:

$\mathrm{IHR}_{p}= \frac{x_{\text{HOSP},p}}{Y_{p}} \mathrm{IIR}_{p}= \frac{x_{\text{ICU,}p}}{Y_{p}}$ $\mathrm{IFR}_{\text{p}}= \frac{x_{\text{DEATH},p}}{Y_{p}}$

where $x_{\text{HOSP},p}$, $x_{\text{ICU},p}$ and $x_{\text{DEATH},p}$ represent the number of COVID-19 cases reported to the national integrated surveillance system during phase $p$ that were hospitalized, admitted to an ICU, or died (Table S3).

**Table S3**. Number of SARS-CoV-2 confirmed infections as reported to the national integrated surveillance system during phase $p$ and those who were admitted to the hospital, to the ICU, or died, respectively, during phase $p$ ^29,34^.

| **Phase** ($p$) | **Dates**  (from – to) | **Estimated infections**  ($Y_{p}$)^†^ | **Confirmed infections**  ($x_{INF,p}$) | **Hospitalized**  ($x_{\text{HOSP},p}$) | **Admitted to ICU**  ($x_{\text{HOSP},p}$) | **Deaths**  ($x_{\text{DEATH},p}$) | |
| --- | --- | --- | --- | --- | --- | --- | --- |
| Ancestral (phase 1) | Feb 21, 2020 - Jun 30, 2020 | 1,659,965  (1,069,842-2,167,503) | 242,700 | 87,467 | 10,419 | 35,874 | |
| Ancestral (phase 2) | Jul 1, 2020 -  Feb 17, 2021 | 6,852,816  (4,384,475-9,101,409) | 2,685,824 | 217,453 | 31,363 | 68,489 | |
| Alpha (phase 3) | Feb 18, 2021 -  Jul 1, 2021 | 6,047,247  (4,246,124-7,785,582) | 1,350,496 | 107,648 | 15,068 | 23,265 | |
| Delta (phase 4) | Jul 2, 2021 -  Dec 23, 2021 | 10,370,333  (6,828,766-14,005,967) | 2,167,897 | 66,387 | 6,766 | 12,205 | |
| Omicron (phase 5) | Dec 24, 2021 - Feb 20, 2022 | 30,609,387  (19,640,697-41,680,099) | 6,332,768 | 71,345 | 4,459 | 14,752 | |
| ^†^ mean and 95%CI | | | | | | |  |

Phase-specific relative reductions in the probability of the different ratios compared to the first ancestral phase were computed as follows:

$$\mathrm{RR}_{\text{IHR},p}=\frac{\mathrm{IHR}_{p}-\mathrm{IHR}_{\text{ancestral (phase 1)}}}{\mathrm{IHR}_{\text{ancestral (phase 1)}}}$$

$$\mathrm{RR}_{\text{IIR},p}=\frac{\mathrm{IIR}_{p}-\mathrm{IIR}_{\text{ancestral (phase 1)}}}{\mathrm{IIR}_{\text{ancestral (phase 1)}}}$$

$$\mathrm{RR}_{\text{IFR},p}=\frac{\mathrm{IFR}_{p}-\mathrm{IFR}_{\text{ancestral (phase 1)}}}{\mathrm{IFR}_{\text{ancestral (phase 1)}}}$$

## 1.4 Model initialization

The population of the model was initialized according to estimates of the Italian population by age at the end of 2020 ^35^ and approximated as constant throughout the two years of simulations. At simulation start, SARS-CoV-2 infection is seeded in a fully susceptible population and the number of initially infectious individuals is determined in such a way to match COVID-19 deaths reported by the surveillance system in the first ancestral phase.

Specifically, to determine the number of initially infectious individuals, we used the following procedure. We considered a grid of candidate seedings ranging from 100 to 10,000 (step of the grid: 200). For each candidate, we distributed initial infections randomly across age-groups and run the model until the end of the first ancestral phase. We inferred the age-specific number of COVID-19 deaths in the first ancestral phase within the model ($M_{a,\text{ancestral (phase 1)}}$), by applying to SARS-CoV-2 infections produced by the model a previously published estimate of the age-specific infection fatality ratio, based on contact tracing data collected in the Lombardy region of Italy between February and April 2020 ^36^. Namely:

$$M_{a,\text{ancestral (phase 1)}}=\mu_{a}y_{a,\text{ancestral (phase 1)}}$$

where $\mu_{a}$ is the age-specific infection fatality ratio estimated for Italy in the first pandemic wave ^36^ and $y_{a,\text{ancestral (phase 1)}}$ is the cumulative number of SARS-CoV-2 infections in the age group $a$ produced by the model in the first ancestral phase.

We chose the candidate seeding of 1,500 initial infections as the one minimizing the root mean square error between the model-estimated number of COVID-19 deaths by age and those reported to the Italian National Integrated Surveillance System during the same period. Estimates of the number of COVID-19 deaths obtained with the selected seeding are shown along with data in Figure S3.


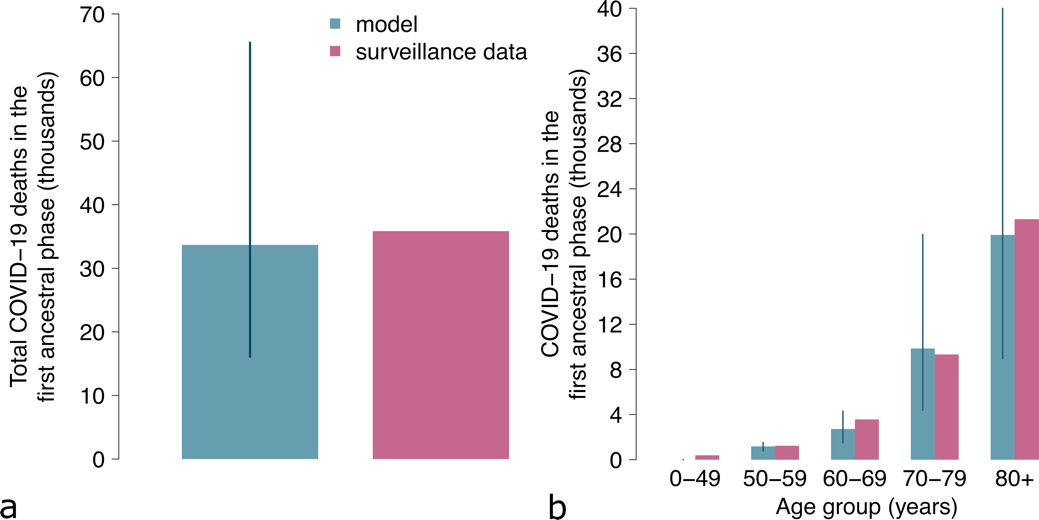


**Figure S3.** **COVID-19 deaths over the first ancestral phase (in thousands).** Blue: mean (bar) and 95% CI (vertical lines) of the model estimates (n = 300 stochastic model realizations); red: data from the Italian Integrated Surveillance System ^29,34^. **a** Total; **b** by age group.

## 1.5 Sensitivity analyses

We assessed the sensitivity of our results considering alternative assumptions on key epidemiological and vaccination parameters on which there is still uncertainty. Table S4 summarizes the parameters varied in the sensitivity analyses considered, together with the baseline values used in the main analysis. In sensitivity A and B, we explored alternative average durations of immunity after natural infection, respectively shorter and longer with respect to the main analysis. In sensitivity C and D, we explored alternative variant-specific average durations of protection after two doses of vaccine and after a booster dose, respectively shorter and longer compared to the main analysis. In sensitivity E, we assumed that infection with previous lineages grant a lower level of cross-protection against re-infection with Omicron BA.1 compared to the main analysis. In sensitivity F, instead of assuming a homogeneous susceptibility across age groups for SARS-CoV-2 variants (i.e. in the Alpha, Delta and Omicron phases), we assumed an age-dependent susceptibility to infection, lower in children and higher for the elderly compared to individuals of working age.

**Table S4**. Description and assumptions on the model parameters that are varied in the sensitivity analyses. Highlighted parameter values are those that are varied with respect to the main analysis.

|  | **Duration of immunity after natural infection**  $1/\upsilon_{R}$ | **Duration of protection from vaccination** | | | **Cross-protection against Omicron BA.1 provided by infection with previous variants**  $\chi_{R_{\text{ancestral}}}\left( t \right);$  $\chi_{R_{\text{alpha}}}\left( t \right);$  $\chi_{R_{\text{delta}}}\left( t \right)$ | **Susceptibility to infection for**  **SARS-CoV-2 variants**  ($r_{a}\left( t \right)$) | **Source** |
| --- | --- | --- | --- | --- | --- | --- | --- |
|  |  | **Two doses** | | **Booster** |  |  |  |
|  |  | $1/\upsilon_{V,\text{delta}}$ | $1/\upsilon_{V,\text{omicron}}$ | $1/\upsilon_{B,\text{omicron}}$ |  |  |  |
| **Main analysis** | 2 years | 200.6 days | 74.5 days | 195.3 days | 0.56 | Homogeneous  $r_{a}\left( t \right)$=1 | See Table S2 |
| **Sensitivity A** | 1 year | 200.6 days | 74.5 days | 195.3 days | 0.56 | Homogeneous  $r_{a}\left( t \right)$=1 | Assumed |
| **Sensitivity B** | 10 years | 200.6 days | 74.5 days | 195.3 days | 0.56 | Homogeneous  $r_{a}\left( t \right)$=1 | Assumed |
| **Sensitivity C** | 2 years | 173.7 days | 64.7 days | 121.1 days | 0.56 | Homogeneous  $r_{a}\left( t \right)$=1 | ^25^ |
| **Sensitivity D** | 2 years | 240 days | 85.5 days | 354.4 days | 0.56 | Homogeneous  $r_{a}\left( t \right)$=1 | ^25^ |
| **Sensitivity E** | 2 years | 200.6 days | 74.5 days | 195.3 days | 0.13 | Homogeneous  $r_{a}\left( t \right)$=1 |  |
| **Sensitivity F** | 2 years | 200.6 days | 74.5 days | 195.3 days | 0.56 | Age-dependent  - $r_{a}\left( t \right)$=0.58 (95%CI 0.34-0.98) under 15y;  - $r_{a}(t)$=1 between 15y and 64y;  - $r_{a}(t)$=1.65 (95%CI 1.03-2.65) over 64y | ^8^ |

# 2. Additional results

## 2.1 Main analysis

We compared the age distribution of SARS-CoV-2 confirmed infections reported to the Italian National Integrated Surveillance system in the different phases considered to that of SARS-CoV-2 infections estimated by the model (Figure S4). Both the data and model estimates suggest a gradual shift of infections towards younger age groups. Estimates of the IHR, IIR and IFR by age show temporal trends compatible with overall estimates, suggesting that the decrease of COVID-19 severity and fatality affected all age groups (Figure S5-S7).


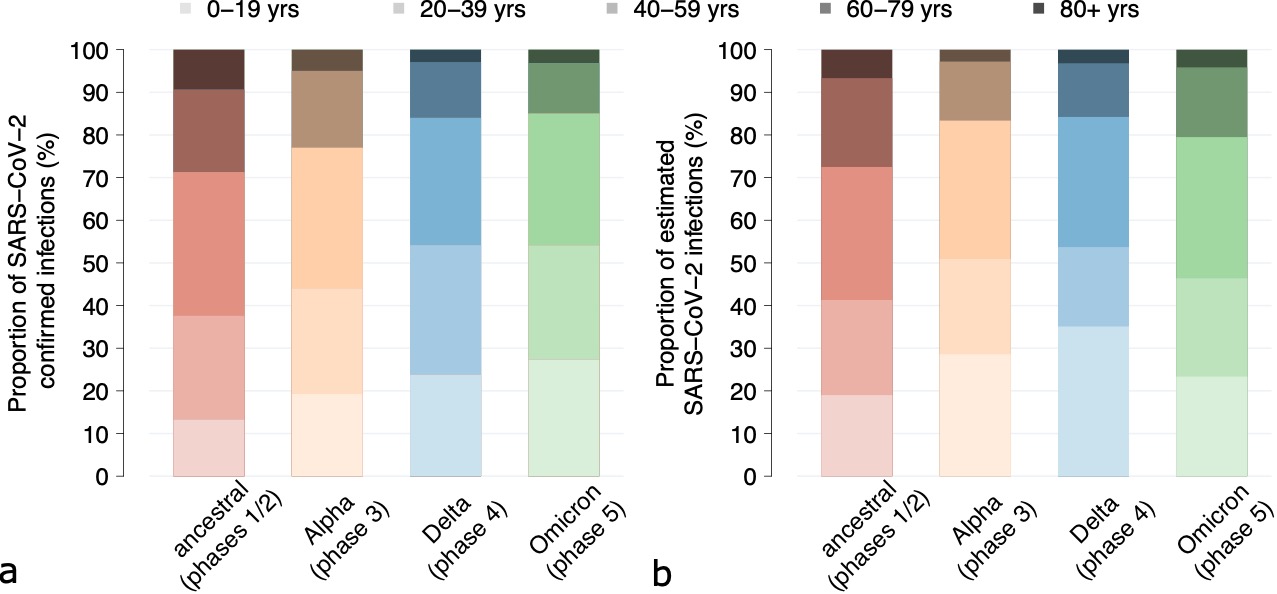


Figure S4. a Age distribution of SARS-CoV-2 confirmed infections reported to the national integrated surveillance system in the ancestral phases (red), in the Alpha phase (orange), in the Delta phase (blue) and in the Omicron phase (green) ^34^. b Mean age distribution of SARS-CoV-2 infections as estimated by the model in the different phases.

**Figure S5.** Changes in SARS-CoV-2 infection hospitalization ratio (IHR) by age group. Bars: mean estimates; vertical lines: 95% CI; n = 300 stochastic model realizations.

**Figure S6.** Changes in SARS-CoV-2 infection ICU ratio (IIR) by age group. Bars: mean estimates; vertical lines: 95% CI; n = 300 stochastic model realizations.

**Figure S7.** Changes in SARS-CoV-2 infection fatality ratio (IFR) by age group. Bars: mean estimates; vertical lines: 95% CI; n = 300 stochastic model realizations.

Figure S8 shows the number of COVID-19 tests (per 1,000 individuals) administered in Italy over time, to assist interpretation of the changing ascertainment ratio over time found by our model. The testing generally increased throughout the pandemic and reached peak values during the Omicron phase, where testing capacity was scaled up by over 3 times compared to a previous peak during the Alpha phase.


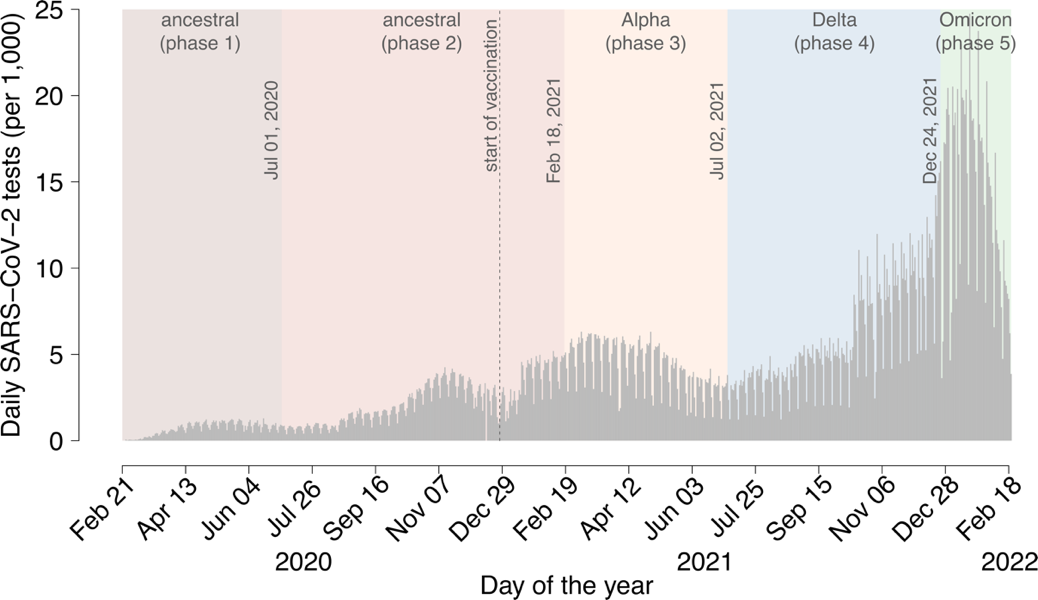


Figure S8. Grey bars represent the daily number of SARS-CoV-2 tests administered per 1,000 individuals ^37^. Background colors indicate the classification in different phases, and the dates indicated within the graph denote the day of transition between consecutive phases. The vertical dotted line denotes the start of the vaccination campaign on December 27, 2020.

We compared the phase-specific percentage of SARS-CoV-2 infections reported to the Italian National Integrated Surveillance system among vaccinated to that estimated by the model (Figure S9). This figure may assist the interpretation of the decrease in the ascertainment ratio found by our model since the Alpha phase. The shift of infection towards vaccinated population segments may have resulted in a higher amount of asymptomatic or pauci-symptomatic infections, which are more difficult to be detected by the surveillance system.


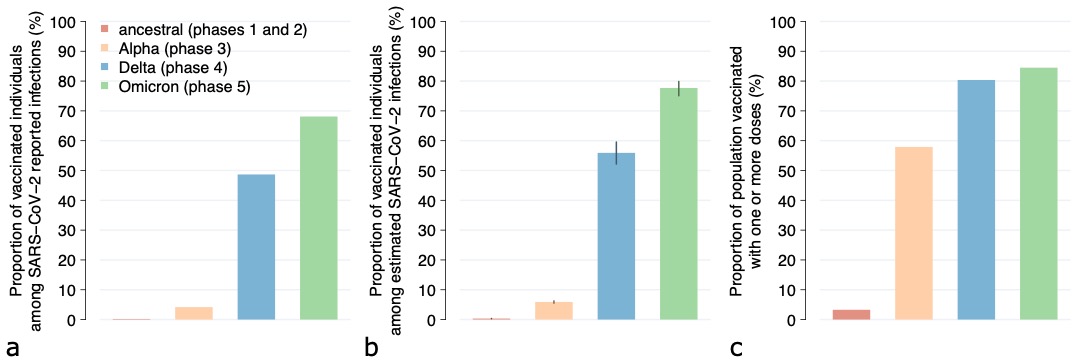


Figure S9. a Proportion of vaccinated individuals (one or more doses, independently from the time at which vaccination was administered) among SARS-CoV-2 confirmed infections reported to the national integrated surveillance system in the ancestral phases (red), in the Alpha phase (orange), in the Delta phase (blue) and in the Omicron phase (green) ^34^. b Proportion of vaccinated individuals (one or more doses, independently from the time at which vaccination was administered) among SARS-CoV-2 infections estimated by the model in the different phases. Bars: mean estimates; vertical lines: 95% CI; n = 300 stochastic model realizations. c Proportion of the Italian population vaccinated with one or more doses at the end of the different phases ^21^.

## 2.2 Sensitivity analyses

We report hereafter results obtained in the sensitivity analyses considered. To ease the comparison with results reported in the main text, we added to the Figures the corresponding mean estimates obtained in the main analysis (marked with black diamonds).

### Duration of immunity after natural infection

We ran two sensitivity analyses assuming that immunity from natural infection lasts shorter or longer compared to the main analysis. Figures S10-S11 show the estimates obtained considering an average duration of immunity after natural infection of 1 year (sensitivity A), while Figures S12-S13 considering an average duration of immunity after natural infection of 10 years (sensitivity B). Results obtained from sensitivity A and sensitivity B suggest that our findings are only marginally affected by the assumption made on this parameter.

Figure S10. SARS-CoV-2 cumulative incidence and infection ascertainment ratio (IAR) as obtained assuming a shorter duration of immunity after natural infection (sensitivity A). a Estimated phase-specific SARS-CoV-2 cumulative incidence (%) as of February 20, 2022, in the overall population and by age classes. Colors indicate the considered phase. Bars: mean estimates; vertical lines: 95% CI; n = 300 stochastic model realizations. b Estimated phase-specific SARS-CoV-2 infection ascertainment ratio between February 21, 2020, and February 20, 2022 (%). Bars: mean estimates; vertical lines: 95% CI; n = 300 stochastic model realizations. Black diamonds represent the mean estimates of the corresponding quantities as obtained from the main analysis.

**Figure S11. Changes in SARS-CoV-2 infection hospitalization ratio, ICU ratio and fatality ratio as obtained assuming a shorter duration of immunity after natural infection (sensitivity A). a** Infection hospitalization ratio (IHR). **b** Infection ICU ratio (IIR). **c** Infection fatality ratio (IFR). **d** Estimated relative reductions in the IHR compared to the first ancestral phase (%). **e** As d but for the IIR. **f** As d but for the IFR. Bars: mean estimates; vertical lines: 95% CI; n = 300 stochastic model realizations. Black diamonds represent the mean estimates of the corresponding quantities as obtained from the main analysis.

Figure S12. SARS-CoV-2 cumulative incidence and infection ascertainment ratio (IAR) as obtained assuming a longer duration of immunity after natural infection (sensitivity B). a Estimated phase-specific SARS-CoV-2 cumulative incidence (%) as of February 20, 2022, in the overall population and by age classes. Colors indicate the considered phase. Bars: mean estimates; vertical lines: 95% CI; n = 300 stochastic model realizations. b Estimated phase-specific SARS-CoV-2 infection ascertainment ratio between February 21, 2020, and February 20, 2022 (%). Bars: mean estimates; vertical lines: 95% CI; n = 300 stochastic model realizations. Black diamonds represent the mean estimates of the corresponding quantities as obtained from the main analysis.

**Figure S13. Changes in SARS-CoV-2 infection hospitalization ratio, ICU ratio and fatality ratio as obtained assuming a longer duration of immunity after natural infection (sensitivity B). a** Infection hospitalization ratio (IHR). **b** Infection ICU ratio (IIR). **c** Infection fatality ratio (IFR). **d** Estimated relative reductions in the IHR compared to the first ancestral phase (%). **e** As d but for the IIR. **f** As d but for the IFR. Bars: mean estimates; vertical lines: 95% CI; n = 300 stochastic model realizations. Black diamonds represent the mean estimates of the corresponding quantities as obtained from the main analysis.

### Duration of protection from vaccination

We ran two sensitivity analyses assuming lower and higher values for the average durations of protection after vaccination. Figures S14-S15 show the estimates obtained assuming shorter durations of vaccine protection compared to the main analysis (sensitivity C), while Figures S16-S17 show the estimates obtained considering longer durations of vaccine protection (sensitivity D). Results obtained from sensitivity C and sensitivity D suggest that the findings are only marginally affected by the assumption made on this parameter.

Figure S14. SARS-CoV-2 cumulative incidence and infection ascertainment ratio (IAR) as obtained assuming a shorter duration of vaccine protection (sensitivity C). a Estimated phase-specific SARS-CoV-2 cumulative incidence (%) as of February 20, 2022, in the overall population and by age classes. Colors indicate the considered phase. Bars: mean estimates; vertical lines: 95% CI; n = 300 stochastic model realizations. b Estimated phase-specific SARS-CoV-2 infection ascertainment ratio between February 21, 2020, and February 20, 2022 (%). Bars: mean estimates; vertical lines: 95% CI; n = 300 stochastic model realizations. Black diamonds represent the mean estimates of the corresponding quantities as obtained from the main analysis.

**Figure S15. Changes in SARS-CoV-2 infection hospitalization ratio, ICU ratio and fatality ratio as obtained assuming a shorter duration of vaccine protection (sensitivity C). a** Infection hospitalization ratio (IHR). **b** Infection ICU ratio (IIR). **c** Infection fatality ratio (IFR). **d** Estimated relative reductions in the IHR compared to the first ancestral phase (%). **e** As d but for the IIR. **f** As d but for the IFR. Bars: mean estimates; vertical lines: 95% CI; n = 300 stochastic model realizations. Black diamonds represent the mean estimates of the corresponding quantities as obtained from the main analysis.

Figure S16. SARS-CoV-2 cumulative incidence and infection ascertainment ratio (IAR) as obtained assuming a longer duration of vaccine protection (sensitivity D). a Estimated phase-specific SARS-CoV-2 cumulative incidence (%) as of February 20, 2022, in the overall population and by age classes. Colors indicate the considered phase. Bars: mean estimates; vertical lines: 95% CI; n = 300 stochastic model realizations. b Estimated phase-specific SARS-CoV-2 infection ascertainment ratio between February 21, 2020, and February 20, 2022 (%). Bars: mean estimates; vertical lines: 95% CI; n = 300 stochastic model realizations. Black diamonds represent the mean estimates of the corresponding quantities as obtained from the main analysis.

**Figure S17. Changes in SARS-CoV-2 infection hospitalization ratio, ICU ratio and fatality ratio as obtained assuming a longer duration of vaccine protection (sensitivity D). a** Infection hospitalization ratio (IHR). **b** Infection ICU ratio (IIR). **c** Infection fatality ratio (IFR). **d** Estimated relative reductions in the IHR compared to the first ancestral phase (%). **e** As d but for the IIR. **f** As d but for the IFR. Bars: mean estimates; vertical lines: 95% CI; n = 300 stochastic model realizations. Black diamonds represent the mean estimates of the corresponding quantities as obtained from the main analysis.

### Cross-protection against Omicron provided by infection with previous variants

Assuming a lower level of cross-protection against Omicron BA.1 results in a minimal shift of infections towards younger age groups in the Omicron phase compared to the main analysis (Figure S18). All other estimates are fully consistent with those obtained in the main analysis (Figure S19).

Figure S18. SARS-CoV-2 cumulative incidence and infection ascertainment ratio (IAR) as obtained assuming that infection with previous lineages grant a lower level of cross-protection against re-infection with Omicron BA.1 (sensitivity E). a Estimated phase-specific SARS-CoV-2 cumulative incidence (%) as of February 20, 2022, in the overall population and by age classes. Colors indicate the considered phase. Bars: mean estimates; vertical lines: 95% CI; n = 300 stochastic model realizations. b Estimated phase-specific SARS-CoV-2 infection ascertainment ratio between February 21, 2020, and February 20, 2022 (%). Bars: mean estimates; vertical lines: 95% CI; n = 300 stochastic model realizations. Black diamonds represent the mean estimates of the corresponding quantities as obtained from the main analysis.

**Figure S19. Changes in SARS-CoV-2 infection hospitalization ratio, ICU ratio and fatality ratio as obtained assuming that infection with previous lineages grant a lower level of cross-protection against re-infection with Omicron BA.1 (sensitivity E). a** Infection hospitalization ratio (IHR). **b** Infection ICU ratio (IIR). **c** Infection fatality ratio (IFR). **d** Estimated relative reductions in the IHR compared to the first ancestral phase (%). **e** As d but for the IIR. **f** As d but for the IFR. Bars: mean estimates; vertical lines: 95% CI; n = 300 stochastic model realizations. Black diamonds represent the mean estimates of the corresponding quantities as obtained from the main analysis.

### Susceptibility to infection for SARS-CoV-2 variants

In this sensitivity analysis, we consider an age-specific susceptibility to SARS-CoV-2 infection for all SARS-CoV-2 lineages (and not only for ancestral lineages, as in the main analysis). Specifically, we assume that, compared to adults between 15 and 64 years, children younger than 15 years of age are less susceptible to infection (relative susceptibility: 0.58 (95%CI 0.34-0.98)^8^), while people above 64 years of age are more susceptible to infection (relative susceptibility: 1.65 (95%CI 1.03-2.65)).

Due to the reduced susceptibility in children and the increased susceptibility in the elderly, results obtained show a shift of infections towards older age groups in the Alpha, Delta and Omicron phases with respect to the main analysis (Figure S20). For instance, in individuals aged 80 years and more, the mean cumulative incidence estimated in the Delta phase is 18.1% (to be compared with 7.4% in the main analysis), while the one estimated in the Omicron phase is 51.1% (to be compared with 28.5% in the main analysis). Despite these differences, the decreasing trend in the SARS-CoV-2 IHR, IIR and IFR is qualitatively and quantitatively consistent with the one obtained in the main analysis (Figure S21).

Figure S20. SARS-CoV-2 cumulative incidence and infection ascertainment ratio (IAR) as obtained by assuming an age-dependent susceptibility for SARS-CoV-2 variants (sensitivity F). a Estimated phase-specific SARS-CoV-2 cumulative incidence (%) as of February 20, 2022, in the overall population and by age classes. Colors indicate the considered phase. Bars: mean estimates; vertical lines: 95% CI; n = 300 stochastic model realizations. b Estimated phase-specific SARS-CoV-2 infection ascertainment ratio between February 21, 2020, and February 20, 2022 (%). Bars: mean estimates; vertical lines: 95% CI; n = 300 stochastic model realizations. Black diamonds represent the mean estimates of the corresponding quantities as obtained from the main analysis.

**Figure S21. Changes in SARS-CoV-2 infection hospitalization ratio, ICU ratio and fatality ratio as obtained by assuming an age-dependent susceptibility for SARS-CoV-2 variants (sensitivity F). a** Infection hospitalization ratio (IHR). **b** Infection ICU ratio (IIR). **c** Infection fatality ratio (IFR). **d** Estimated relative reductions in the IHR compared to the first ancestral phase (%). **e** As d but for the IIR. **f** As d but for the IFR. Bars: mean estimates; vertical lines: 95% CI; n = 300 stochastic model realizations. Black diamonds represent the mean estimates of the corresponding quantities as obtained from the main analysis.

# References

1. Marziano V, Guzzetta G, Mammone A, et al. The effect of COVID-19 vaccination in Italy and perspectives for living with the virus. *Nat Commun*. 2021;12(1):7272. doi:10.1038/s41467-021-27532-w

2. Yang J, Marziano V, Deng X, et al. Despite vaccination, China needs non-pharmaceutical interventions to prevent widespread outbreaks of COVID-19 in 2021. *Nat Hum Behav*. 2021;5(8):1009-1020. doi:10.1038/s41562-021-01155-z

3. Trentini F, Marziano V, Guzzetta G, et al. Pressure on the Health-Care System and Intensive Care Utilization During the COVID-19 Outbreak in the Lombardy Region of Italy: A Retrospective Observational Study in 43,538 Hospitalized Patients. *Am J Epidemiol*. 2022;191(1):137-146. doi:10.1093/aje/kwab252

4. Guzzetta G, Riccardo F, Marziano V, et al. Impact of a Nationwide Lockdown on SARS-CoV-2 Transmissibility, Italy. *Emerg Infect Dis*. 2021;27(1):267-270. doi:10.3201/eid2701.202114

5. Manica M, Guzzetta G, Riccardo F, et al. Impact of tiered restrictions on human activities and the epidemiology of the second wave of COVID-19 in Italy. *Nat Commun*. 2021;12(1):4570. doi:10.1038/s41467-021-24832-z

6. Istituto Superiore di Sanità. Monitoraggio delle varianti del virus SARS-CoV-2 di interesse in sanità pubblica in Italia. https://www.epicentro.iss.it/coronavirus/sars-cov-2-monitoraggio-varianti-indagini-rapide

7. Mossong J, Hens N, Jit M, et al. Social Contacts and Mixing Patterns Relevant to the Spread of Infectious Diseases. *PLOS Med*. 2008;5(3):e74. doi:10.1371/journal.pmed.0050074

8. Hu S, Wang W, Wang Y, et al. Infectivity, susceptibility, and risk factors associated with SARS-CoV-2 transmission under intensive contact tracing in Hunan, China. *Nat Commun*. 2021;12(1):1533. doi:10.1038/s41467-021-21710-6

9. Harris RJ, Hall JA, Zaidi A, Andrews NJ, Dunbar JK, Dabrera G. Effect of Vaccination on Household Transmission of SARS-CoV-2 in England. *N Engl J Med*. 2021;385(8):759-760. doi:10.1056/NEJMc2107717

10. Lipsitch M, Kahn R. Interpreting vaccine efficacy trial results for infection and transmission. *Vaccine*. 2021;39(30):4082-4088. doi:10.1016/j.vaccine.2021.06.011

11. Hall VJ, Foulkes S, Charlett A, et al. SARS-CoV-2 infection rates of antibody-positive compared with antibody-negative health-care workers in England: a large, multicentre, prospective cohort study (SIREN). *The Lancet*. 2021;397(10283):1459-1469. doi:10.1016/S0140-6736(21)00675-9

12. Altarawneh HN, Chemaitelly H, Hasan MR, et al. Protection against the Omicron Variant from Previous SARS-CoV-2 Infection. *N Engl J Med*. 2022;386(13):1288-1290. doi:10.1056/NEJMc2200133

13. Andrews N, Tessier E, Stowe J, et al. Duration of Protection against Mild and Severe Disease by Covid-19 Vaccines. *N Engl J Med*. 2022;386(4):340-350. doi:10.1056/NEJMoa2115481

14. Fabiani M, Puopolo M, Morciano C, et al. Effectiveness of mRNA vaccines and waning of protection against SARS-CoV-2 infection and severe covid-19 during predominant circulation of the delta variant in Italy: retrospective cohort study. *BMJ*. 2022;376:e069052. doi:10.1136/bmj-2021-069052

15. Andrews N, Stowe J, Kirsebom F, et al. Covid-19 Vaccine Effectiveness against the Omicron (B.1.1.529) Variant. *N Engl J Med*. 2022;386(16):1532-1546. doi:10.1056/NEJMoa2119451

16. Fabiani M, Puopolo M, Filia A, et al. Effectiveness of an mRNA vaccine booster dose against SARS-CoV-2 infection and severe COVID-19 in persons aged ≥60 years and other high-risk groups during predominant circulation of the delta variant in Italy, 19 July to 12 December 2021. *Expert Rev Vaccines*. 2022;0(0):1-8. doi:10.1080/14760584.2022.2064280

17. Svensson A. A note on generation times in epidemic models. *Math Biosci*. 2007;208(1):300-311. doi:10.1016/j.mbs.2006.10.010

18. Cereda D, Manica M, Tirani M, et al. The early phase of the COVID-19 epidemic in Lombardy, Italy. *Epidemics*. 2021;37:100528. doi:10.1016/j.epidem.2021.100528

19. Manica M, Litvinova M, De Bellis A, et al. *Estimation of the Incubation Period and Generation Time of SARS-CoV-2 Alpha and Delta Variants from Contact Tracing Data*. arXiv; 2022. doi:10.48550/arXiv.2203.07063

20. Manica M, Bellis AD, Guzzetta G, et al. Intrinsic generation time of the SARS-CoV-2 Omicron variant: An observational study of household transmission. *Lancet Reg Health – Eur*. 2022;19. doi:10.1016/j.lanepe.2022.100446

21. Covid-19 Opendata Vaccini. https://github.com/italia/covid19-opendata-vaccini

22. World Health Organization. *WHO SAGE Roadmap for Prioritizing the Use of COVID-19 Vaccines in the Context of Limited Supply: An Approach to Inform Planning and Subsequent Recommendations Based upon Epidemiologic Setting and Vaccine Supply Scenarios, 13 November 2020*. Version 1.1. World Health Organization; 2020. https://apps.who.int/iris/handle/10665/341448

23. Dagan N, Barda N, Kepten E, et al. BNT162b2 mRNA Covid-19 Vaccine in a Nationwide Mass Vaccination Setting. *N Engl J Med*. 2021;384(15):1412-1423. doi:10.1056/NEJMoa2101765

24. Polack FP, Thomas SJ, Kitchin N, et al. Safety and Efficacy of the BNT162b2 mRNA Covid-19 Vaccine. *N Engl J Med*. 2020;383(27):2603-2615. doi:10.1056/NEJMoa2034577

25. Menegale F, Manica M, Zardini A, et al. Evaluation of Waning of SARS-CoV-2 Vaccine–Induced Immunity: A Systematic Review and Meta-analysis. *JAMA Netw Open*. 2023;6(5):e2310650. doi:10.1001/jamanetworkopen.2023.10650

26. Diekmann O, Heesterbeek JAP, Metz JAJ. On the definition and the computation of the basic reproduction ratio R0 in models for infectious diseases in heterogeneous populations. *J Math Biol*. 1990;28(4):365-382. doi:10.1007/BF00178324

27. Diekmann O, Heesterbeek J a. P, Roberts MG. The construction of next-generation matrices for compartmental epidemic models. *J R Soc Interface*. 2010;7(47):873-885. doi:10.1098/rsif.2009.0386

28. Marziano V, Poletti P, Trentini F, Melegaro A, Ajelli M, Merler S. Parental vaccination to reduce measles immunity gaps in Italy. *eLife*. 2019;8:e44942. doi:10.7554/eLife.44942

29. Riccardo F, Ajelli M, Andrianou XD, et al. Epidemiological characteristics of COVID-19 cases and estimates of the reproductive numbers 1 month into the epidemic, Italy, 28 January to 31 March 2020. *Eurosurveillance*. 2020;25(49):2000790. doi:10.2807/1560-7917.ES.2020.25.49.2000790

30. Task force COVID-19 del Dipartimento Malattie Infettive, Servizio di Informatica, Istituto Superiore di Sanità. *Epidemia COVID-19. Aggiornamento Nazionale: 2 Marzo 2022*. https://www.epicentro.iss.it/coronavirus/bollettino/Bollettino-sorveglianza-integrata-COVID-19_2-marzo-2022.pdf

31. Cori A, Ferguson NM, Fraser C, Cauchemez S. A New Framework and Software to Estimate Time-Varying Reproduction Numbers During Epidemics. *Am J Epidemiol*. 2013;178(9):1505-1512. doi:10.1093/aje/kwt133

32. Thompson RN, Stockwin JE, van Gaalen RD, et al. Improved inference of time-varying reproduction numbers during infectious disease outbreaks. *Epidemics*. 2019;29:100356. doi:10.1016/j.epidem.2019.100356

33. Marziano V. Code and data of article “Estimating SARS-CoV-2 infections and associated changes in COVID-19 severity and fatality.” Published online June 5, 2023. doi:10.5281/zenodo.8006661

34. Istituto Superiore di Sanità. *COVID-19 ISS Open Data – EpiCentro.* https://www.epicentro.iss.it/coronavirus/open-data/covid_19-iss.xlsx

35. Italian National Institute of Statistics (ISTAT). Popolazione residente al 1° Gennaio 2021 per età, sesso e stato civile. https://demo.istat.it/popres/index.php?anno=2021&lingua=ita

36. Poletti P, Tirani M, Cereda D, et al. Age-specific SARS-CoV-2 infection fatality ratio and associated risk factors, Italy, February to April 2020. *Eurosurveillance*. 2020;25(31):2001383. doi:10.2807/1560-7917.ES.2020.25.31.2001383

37. Dati COVID-19 Italia. Published online May 19, 2022. https://github.com/pcm-dpc/COVID-19
